# Mitochondrial permeability transition pore induces mitochondria injury in Huntington disease

**DOI:** 10.1186/1750-1326-8-45

**Published:** 2013-12-11

**Authors:** Rodrigo A Quintanilla, Youngnam N Jin, Rommy von Bernhardi, Gail VW Johnson

**Affiliations:** 1 Department of Anesthesiology, University of Rochester Medical Center, 601 Elmwood Ave, Box 604 (for courier: Rm 4-6314), Rochester, NY 14642, USA; 2 Laboratorio de Neurociencias, Departamento de Neurología, Escuela de Medicina, Pontificia Universidad Católica de Chile, Marcoleta 391, Santiago, Chile

**Keywords:** Huntington’s disease, Huntingtin, Mitochondria dysfunction, Oxidative stress, Mitochondria permeability transition pore, Striatal cells, Mitochondrial fragmentation

## Abstract

**Background:**

Mitochondrial impairment has been implicated in the pathogenesis of Huntington’s disease (HD). However, how mutant huntingtin impairs mitochondrial function and thus contributes to HD has not been fully elucidated. In this study, we used striatal cells expressing wild type (STHdh^Q7/Q7^) or mutant (STHdh^Q111/Q111^) huntingtin protein, and cortical neurons expressing the exon 1 of the huntingtin protein with physiological or pathological polyglutamine domains, to examine the interrelationship among specific mitochondrial functions.

**Results:**

Depolarization induced by KCl resulted in similar changes in calcium levels without compromising mitochondrial function, both in wild type and mutant cells. However, treatment of mutant cells with thapsigargin (a SERCA antagonist that raises cytosolic calcium levels), resulted in a pronounced decrease in mitochondrial calcium uptake, increased production of reactive oxygen species (ROS), mitochondrial depolarization and fragmentation, and cell viability loss. The mitochondrial dysfunction in mutant cells was also observed in cortical neurons expressing exon 1 of the huntingtin protein with 104 Gln residues (Q104-GFP) when they were exposed to calcium stress. In addition, calcium overload induced opening of the mitochondrial permeability transition pore (mPTP) in mutant striatal cells. The mitochondrial impairment observed in mutant cells and cortical neurons expressing Q104-GFP was prevented by pre-treatment with cyclosporine A (CsA) but not by FK506 (an inhibitor of calcineurin), indicating a potential role for mPTP opening in the mitochondrial dysfunction induced by calcium stress in mutant huntingtin cells.

**Conclusions:**

Expression of mutant huntingtin alters mitochondrial and cell viability through mPTP opening in striatal cells and cortical neurons.

## Background

Huntington disease (HD) is a neurodegenerative disease caused by the pathological elongation of the CAG repeats in exon 1 of the huntingtin protein gene [1]. HD is characterized by dysfunction and loss of striatal neurons in the initial stages, subsequently involving the cortex and other brain regions in later stages of the disease [2]. Expression of mutant huntingtin can result in transcriptional deregulation [3], and proteasome dysfunction in brain cells [4-6]. More importantly, calcium deregulation [7,8], and mitochondrial dysfunction [9-11] have been strongly implicated in the pathogenesis of HD.

Regarding mitochondrial dysfunction, previous studies have shown impairment of mitochondrial complex activities, which were specifically observed in the striatum of HD patients [12,13]. Studies in primary neurons from yeast artificial chromosome 128 (YAC128) mice, a transgenic mouse model of HD that expresses the full-length human HD gene with several CAG repeats [14], indicated that robust NMDAR activation produced mitochondrial dysfunction and inability to regulate cytosolic calcium homeostasis in medium-sized spiny neurons (MSNs) [15]. In addition, functional experiments, using immortalized striatal cell lines (STHdh cells) and primary striatal neurons from YAC128 HD mice expressing mutant huntingtin, revealed impairments in mitochondrial calcium handling [16]. More importantly, previous studies showed that mutant huntingtin expression impaired mitochondrial ATP production [17], increased sensitivity of mitochondria to calcium-induced respiratory defects [18], and induced mitochondrial damage [19]. Studies in isolated mitochondria showed that mitochondrial dysfunction induced by calcium stress can be prevented by cyclosporine A (CsA), a blocker of mitochondrial permeability transition pore (mPTP) induction [18,20], with immunosuppressant effect [18]. Altogether these are important observations but the use of isolated mitochondria limits their physiological relevance. Therefore, new studies of mitochondrial function in intact striatal cells that express mutant huntingtin are needed to move forward in our understanding of the pathogenesis of HD.

Interestingly, there is data suggesting that mutant huntingtin impairs mitochondrial morphology and trafficking [21-24]. Overexpression of huntingtin proteins containing polyglutamine repeats in the pathological range, but not those in the non-pathological range, increases oxidative stress-induced mitochondrial fragmentation in HeLa cells [22]. In STHdh^Q111/Q111^ cells and in lymphoblasts from HD patients, significant mitochondrial fragmentation was observed with cristae alterations that were aggravated by stimulation of apoptosis [25]. Interestingly, a recent study showed evidence of mitochondrial fragmentation with increased expression of dynamin-related protein 1 (Drp1) and mitochondrial fission protein 1 (Fis1), which control mitochondrial fission in cerebral cortical samples from HD patients [26]. However, the effects of calcium stress on mitochondrial fragmentation in mutant huntingtin cells needs to be examined.

In this work to study the mechanisms by which mutant huntingtin affects mitochondrial function (calcium homeostasis, reactive oxygen species (ROS) production, and morphology) in response to calcium stress in a cellular contex, we used cell lines obtained from wild type (Hdh^Q7/Q7^) and mutant huntingtin knock-in (Hdh^Q111/Q111^) mice [27] and rat cortical neurons. We examined cytosolic and mitochondria calcium homeostasis, mitochondrial function, and mitochondrial morphology in striatal cells that expressed wild type (STHdh^Q7/Q7^) and mutant huntingtin (STHdh^Q111/Q111^). Cells were treated with different stressors that increased cytosolic calcium concentration by different mechanisms. Depolarization by KCl increased cytosolic and mitochondrial calcium levels with no apparent differences between wild type and mutant cells. In wild type and mutant cells treatment with thapsigargin induced a two-fold increase in cytosolic calcium levels compared with KCl. However, in cells expressing mutant huntingtin, thapsigargin-induced increase in cytosolic calcium affected mitochondrial health, resulting membrane potential loss, oxidative stress, and mitochondrial fragmentation. More importantly, these changes were prevented by pre-treatment with cyclosporine A (CsA) but no by FK-506, an inhibitor of calcineurin (a calcium dependent phosphatase). In addition, thapsigargin induced the opening of mPTP in mutant huntingtin cells. Expression of a mutant huntingtin construct in cortical neurons also resulted in calcium stress-induced loss of mitochondrial membrane potential, which was also prevented by CsA.

## Results

### Effect of depolarization on calcium regulation in mutant huntingtin cells

**Figure 1:**
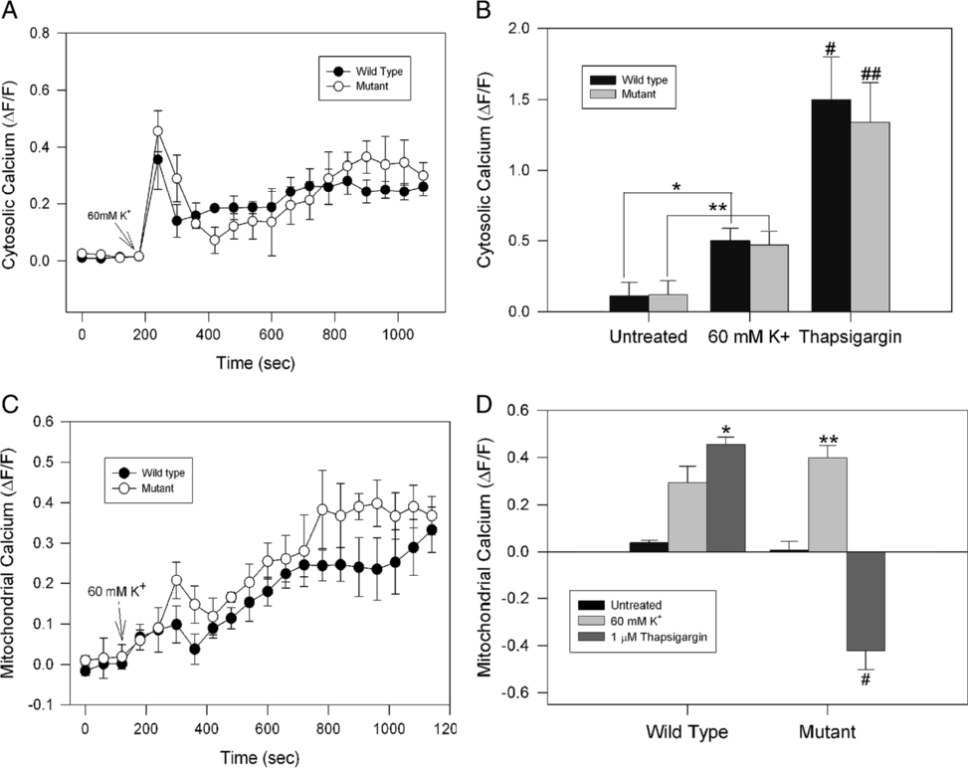
**Effect of cell depolarization on calcium regulation in striatal cells. A, B** striatal cells were loaded with Fluo3 AM and cytosolic calcium was measured using confocal microscopy. Fluo3 fluorescence changes were measured after cells were treated with 60 mM KCl for 30 min. Fluo3 intensity variations represent changes in cytosolic calcium levels and are expressed using the pseudo ratio ΔF/F_0_ (see Methods). **B**, quantitative analysis of 4 independent experiments of striatal cells treated with 60 mM KCl and 1 μM thapsigargin. No differences in cytosolic calcium peak were observed when wild type and mutant cells were compared. Data correspond to the mean ± S.E.M. of four independent experiments. * *p* < 0.05 compared with untreated wild type cells, ** *p < 0.05* compared with untreated mutant cells, # *p* < 0.05 compared to with wild type cells treated with KCL, and ## *p* < 0.05 compared to mutant cells treated with KCL. **C**, **D** clonal striatal cells were loaded with Rhod2 AM/MitoGreen and treated with 60 mM KCl during 30 min for mitochondrial calcium determinations using confocal microscopy. KCl induced a mitochondrial calcium increase in both cell types, with no significant differences between wild type and mutant cells. Mitochondrial calcium levels were represented using pseudo ratio ΔF/F_0_. **D**, quantitative analysis of 4 independent experiments shows an increased mitochondrial calcium levels in both mutant and wild type cells exposed to KCl. Interestingly, mutant cells treated with 1 μM thapsigargin for 30 min showed a severe decrease in mitochondrial calcium uptake compared with wild type cells. Data correspond to the mean ± S.E.M. of four independent experiments. * *p* < 0.05 compared with wild type cells treated with KCl, ** *p* < 0.05 compared with mutant cells treated with KCl, and # *p* < 0.05 compared to mutant cells treated with KCL.

It has been suggested that mutant huntingtin induces calcium-handling defects in neuronal cells exposed to various stressors [28]. However, it is unclear which mechanisms are involved in this deregulation. Therefore, we evaluated the cytosolic and mitochondrial calcium changes in clonal striatal cells expressing mutant huntingtin protein in response to depolarization and calcium overload. Interestingly, immunofluorescence studies of striatal cells stained with an anti-huntingtin antibody and mitochondrial markers (Mitotracker® Red CM-H_2_XRos (MitoRed) and cytochrome C) showed an evident colocalization, indicating that huntingtin and mitochondria are associated (Additional file 1: Figure S1). To evaluate calcium changes in response to depolarization, wild type and mutant striatal cells were treated with 60 mM KCl, which results in a transient intra-neuronal calcium increase [29]. Cytosolic and mitochondrial calcium levels were simultaneously monitored using Fluo-3AM and Rhod-2AM, respectively [19]. As expected, KCl-induced depolarization produced a transient increase in cytosolic and mitochondrial calcium levels that was similar in both cell types (Figure 1A, C). Thapsigargin was used to evaluate the effects of pathological cytosolic calcium increases on mitochondrial function. This compound inhibits calcium uptake by endoplasmic reticulum and thus results in an abnormaly high increase of intracellular calcium [19]. To compare the cytosolic calcium response, we quantified the peak of cytosolic calcium increase induced by 60 mM KCl or 1 μM thapsigargin for 30 min (Figure 1B). Representative confocal images and trends of cytosolic calcium levels produced by thapsigargin showed a rapid and acute increase in calcium concentration which returned to basal levels after 2 min (Additional file 2: Figure S2A and B). Treatment with thapsigargin clearly resulted in an approximately a 3-fold increase in cytosolic calcium levels compared with the effect of KCl, in both cell types (Figure 1B). Additional studies with other calcium mobilizing agents; 4-BrA23187 plus 6 mM Ca^2+^ and ionomycin, revealed no differences in peaks of cytosolic calcium between wild and mutant cells (Additional file 2: Figure S2C). However, treatment with 4-BrA23187 (1nM) plus 6mM Ca^2+^ induced a significantly greater increase in cytosolic calcium levels compared to KCl treatment (Additional file 2: Figure S2C). In addition, we evaluated mitochondrial calcium levels in wild type and mutant huntingtin expressing cells exposed to KCl and thapsigargin during 30 min (Figure 1C, D). KCl produced a consistent increase in mitochondrial calcium levels, with a similar pattern in wild type and mutant cells (Figure 1C). In contrast, calcium overload induced by thapsigargin resulted in a significant decrease in mitochondrial calcium uptake in mutant cells, whereas wild type cells showed a transient increase in mitochondrial calcium (Figure 1C, D). Quantitated data of striatal cells treated with thapsigargin for 30 min consistently showed that the abnormal increase in cytosolic calcium deregulated mitochondrial calcium uptake in striatal cells expressing mutant huntingtin (Figure 1D). In addition, for all studies presented in this paper, vehicle studies were made. Vehicle treatments were not included because they did not show significant changes compared to untreated cells.

### Cyclosporine A prevented mitochondrial depolarization induced by thapsigargin but not by H_2_O_2_ in mutant huntingtin cells

**Figure 2:**
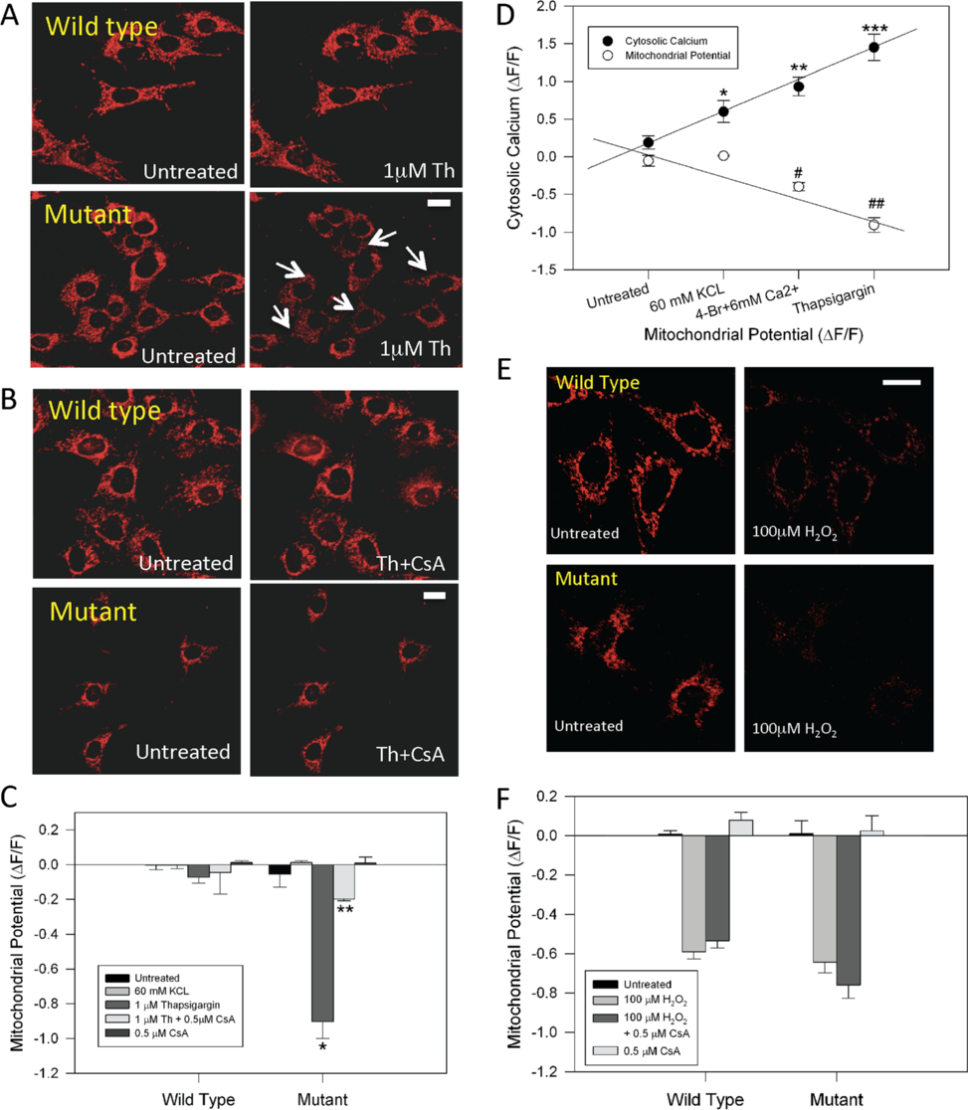
**Treatment with Cyclosporine A prevents mitochondrial injury induced by thapsigargin in mutant HD cells. A**, live confocal images of MitoRed, an indicator of mitochondrial potential, in striatal cells, untreated and treated with 1 μM thapsigargin (Th) for 30 min. Thapsigargin induces a significant decrease in the mitochondrial potential in mutant cells (see white arrows). Bar represents 10 μm. **B**, images show that CsA protects mutant huntingtin cells exposed to thapsigargin from mitochondrial impairment. Bar represents 10 μm. **C**, quantification of MitoRed intensities as relative units (ΔF/F_0_) in striatal cells treated for 30 min with experimental conditions as indicated. Data are the mean ± S.E.M. of 4 independent experiments. *, *p* < 0.05 compared with wild type cells treated with thapsigargin; ** *p* < 0.05 compared with mutant cells exposed to thapsigargin. **D**, correlation analysis of mitochondrial potential and cytosolic calcium observed in mutant cells treated with the indicated conditions for 30 min. Cytosolic calcium was estimated from the peak levels. Mitochondrial potential were obtained after 30 min of treatment for every condition. Data are expressed as the mean ± S.E.M. of 4 independent experiments. *, *p* < 0.05 compared to control; ** *p* < 0.05 compared to 60 mM KCL; ***, p < 0.05 compared to 4-BrA23187(1 nM) + 6 mM Ca^2+.^ # compared to 60 mM KCL; ## compared to 4-BrA23187 + 6mM Ca^2+^. **E**, confocal images of mitochondrial potential in striatal cells, untreated and treated with 100 μM H_2_O_2_ for 1h. Bar represents 10 μm. **F**, striatal cells were incubated with 100 μM H_2_O_2_ for 1 h and mitochondrial potential was evaluated. MitoRed levels are show as relative units (ΔF/F_0_) at 1 h. Data is expressed as the mean ± S.E.M. of 3 independent experiments.

There is evidence that pathological cytosolic calcium increase can activate the mitochondrial permeability transition pore (mPTP) [30]. In general, the opening of mPTP has been associated with the collapse of mitochondrial membrane potential, complex I deficiency and changes in free radical levels, and is thought to be a pathologic condition involved in cell injury [31-33]. Therefore, we evaluated mitochondrial membrane potential changes in striatal cells exposed to thapsigargin, with or without treatment with 0.5 μM cyclosporine A (CsA), and inhibitor of mPTP opening [30] for 2 h (Figure 2A, B, and C). Figure 2A and B shows representative confocal images of striatal cells loaded with MitoRed, a mitochondrial potential indicator, and exposed to 1 μM thapsigargin with or without pretreatment with 0.5 μM CsA. Previously, we have validated the use of this mitochondrial potential dye by comparing it to tetramethylrhodamine methyl ester (TMRE), a very well established mitochondrial potential dye in clonal striatal cells [18,19]. These studies demonstrated that treatment of both wild type and mutant cells with the mitochondrial uncoupler trifluorocarbonylcyanide phenylhydrazone (FCCP) (10 μM) resulted in a similar robust decrease in MitoRed and TMRE fluorescence, indicating a loss of mitochondrial potential [18]. Treatment of mutant cells for 30 min with thapsigargin resulted in mitochondrial membrane depolarization, an effect that was not observed in wild type cells (Figure 2A). Pre-treatment with CsA for two hours prevented mitochondrial impairment induced by thapsigargin in mutant cells (Figure 2B). Quantitative analysis of striatal cells treated with 60 mM KCl for 30 min, 1 μM thapsigargin for 30 min, 1 μM thapsigargin + pretreatment with 0.5 μM CsA for 2 h, or pretreatment with 0.5 μM CsA for 2 h, showed that CsA inhibited by nearly 80% the reduction of mitochondrial potential induced by thapsigargin in mutant cells (Figure 2C). Noteworthy, CsA did not induce significant changes in mitochondrial membrane potential of wild type and mutant cells treated with 60 mM KCl for 30 min (Figure 2C). However, thapsigargin treatment significantly affected mitochondrial function in mutant huntingtin expressing cells, indicating that high cytosolic calcium levels mediated the mitochondrial impairment observed in mutant cells. Interestingly, correlation analysis of cytosolic calcium and mitochondrial potential levels in striatal cells under the indicated conditions showed that mitochondrial depolarization had a strong dependency on the calcium concentration in mutant huntingtin cells (Figure 2D). In addition, mitochondrial potential loss was not present in mutant cells treated with KCl, but it was observed in cells treated with 1 nM 4-BrA23187+ 6 mM Ca^2+^, which significantly increased cytosolic calcium compared to KCl (Additional file 2: Figure S2), showed a mild but significant mitochondrial depolarization and decreased mitochondrial calcium uptake compared with wild type cells (Additional file 3: Figure S3). Further, we have previously reported that treatment with 1nM 4-BrA23187 did not induce significant changes in mitochondrial potential, mitochondrial calcium uptake, and cell viability [18].

Accumulative evidence suggests that mPTP could be activated in response to calcium stress generating mitochondrial depolarization, mitochondrial calcium defects and reduced ATP production [30,34]. Oxidative stress has been involved in the pathogenesis of HD [17,24]. It is postulated that mutant huntingtin interferes with transcriptional processes, leading to disruption of the expression of genes involved in ROS response rather than direct mitochondrial damage mediated by calcium disturbances [17,24]. Therefore, we evaluated mitochondrial potential levels in striatal cells exposed to an oxidant agent (Figure 2E). Treatment with 100 μM H_2_O_2_ for 30 min resulted in a robust reduction of mitochondrial potential in both wild type and mutant cells (Figure 2D). Interestingly, pretreatment with 0.5 μM CsA did not prevent mitochondrial potential loss induced by H_2_O_2_, indicating that mPTP did not participate in mitochondrial impairment induced by H_2_O_2_ in striatal cells. In conclusion, these results suggest a role for mPTP on mitochondrial damage triggered by a pathological calcium increase in mutant huntingtin cells.

### Effect of FK-506 on thapsigargin-induced mitochondrial impairment in mutant huntingtin cells

**Figure 3:**
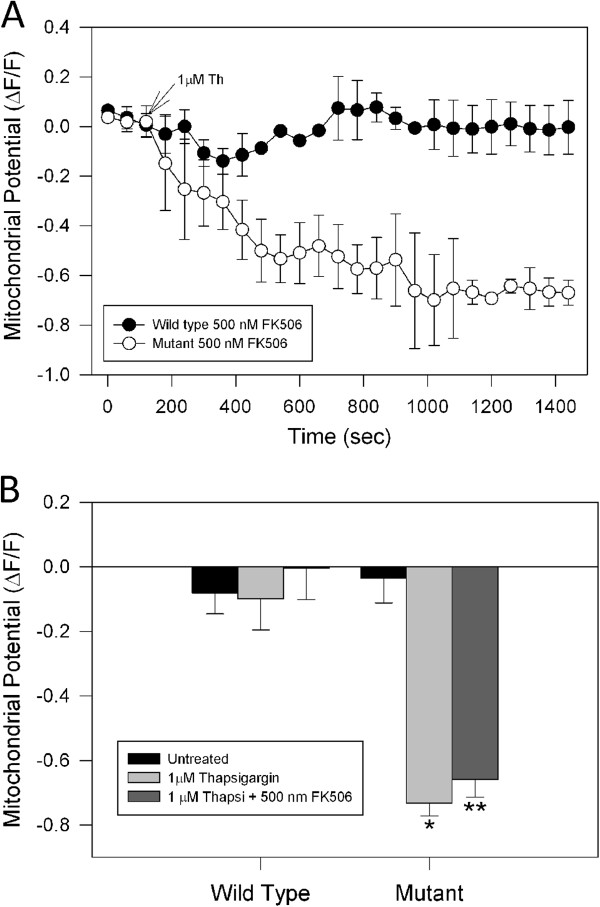
**Effects of FK-506 on mitochondria impairment induced by thapsigargin in mutant huntingtin cells. A**, wild type and mutant cells were incubated with 500 nM FK-506, an inhibitor of calcineurin activity, for 2 h prior to mitochondrial potential determinations using MitoRed. Striatal cells loaded with MitoRed were exposed to 1 μM thapsigargin (Th) for 30 min, and mitochondrial potential was evaluated using confocal microscopy. Treatment with thapsigargin reduced mitochondrial potential levels in mutant striatal cells pre-treated with FK-506, indicating that calcineurin activity was not playing a role in mitochondrial impairment induced by calcium stress in mutant huntingtin cells. **B**, quantitation of MitoRed intensities, which represents mitochondrial potential levels, as relative units at 30 min (ΔF/F_0_) in striatal cells treated with conditions indicated. Data are expressed as the mean ± S.E.M. of 4 independent experiments. *, *p* < 0.05 compared with wild type cells treated with thapsigargin; ** *p* < 0.05 compared with wild type cells treated with thapsigargin.

It has been reported that CsA could also inhibits the activity of the phosphatase, calcineurin [35]. Therefore, to determine the specificity of CsA for blocking the mPTP and if the calcineurin pathway was involved in the mitochondrial dysfunction in mutant cells, we pre-treated wild type and mutant cells with FK-506, which blocks calcineurin, but lacks effect on mPTP [35,36]. Treatment with 500 nM FK-506 for 2 h had no effect on mitochondrial impairment induced by thapsigargin in mutant striatal cells (Figure 3A, B), suggesting that CsA specifically depended on its inhibition of mPTP in mutant huntingtin cells.

### Cyclosporine A partially restored the loss of mitochondrial calcium uptake induced by thapsigargin in mutant cells

**Figure 4:**
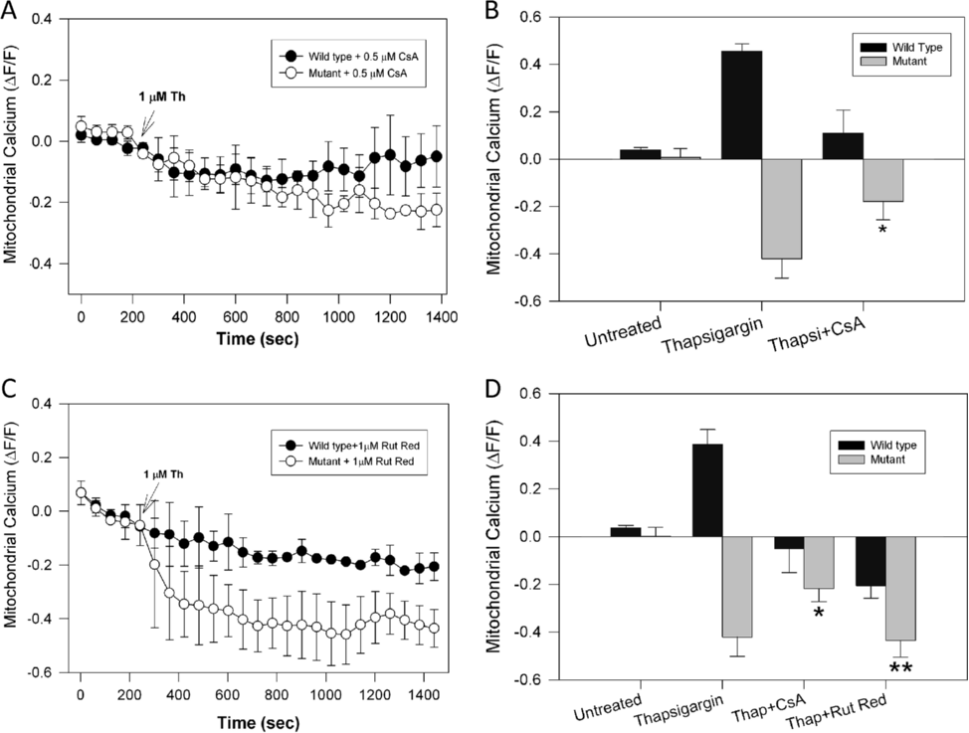
**Treatment with Cyclosporine A improved mitochondrial calcium uptake in mutant huntingtin cells exposed to calcium overload. A**, striatal cells were loaded with Rhod2 AM dye to determine mitochondrial calcium levels in cells exposed to 1 μM thapsigargin (Th) during 30 min. Thapsigargin treatment severely decreased mitochondrial calcium uptake. Striatal cells were incubated for 2 h with 0.5 μM Cyclosporine A (CsA) prior to determination of mitochondrial calcium levels in response to thapsigargin treatment. CsA prevented the mitochondrial calcium decrease induced by thapsigargin in the mutant cells. **B**, quantification of mitochondrial calcium levels from 4 independent experiments obtained from cells exposed to thapsigargin for 30 min. *, *p* < 0.05 compared with mutant cells treated with thapsigargin alone. **C**, wild type and mutant cells were pre-treated with 1 μM ruthenium red for 1 h, before addition of 1 μM thapsigargin. Ruthenium red did not prevent the mitochondrial calcium decrease induced by thapsigargin in mutant cells. **D**, quantification of mitochondrial calcium uptake at 30 min shows that thapsigargin only induced a significant increase in mitochondrial calcium only in wild type cells. Thapsigargin treatment of mutant cells resulted in a pronounced decrease in mitochondrial calcium uptake by mutant cells, and this event was partially blocked by CsA pre-treatment. Data are the mean ± S.E.M. of 3 separate experiments. *, *p* < 0.05 compared with mutant cells treated with thapsigargin; **, *p* < 0.05 compared with mutant cells treated with thapsigargin plus CsA. *p* < 0.05 for nonpaired Student’s *t* test.

Loss of mitochondrial potential could affect mitochondrial calcium uptake and mitochondrial ROS production [19]. In our model, calcium increases induced by 4-BrA123 and thapsigargin induced mitochondrial depolarization and compromised mitochondrial calcium uptake in mutant huntingtin cells (Figure 2 and Additional file 3: Figure S3) [19]. To determine if the opening of mPTP is involved in the inhibition of calcium uptake by the mitochondria in mutant cells, striatal cells were pretreated with 0.5 μM CsA for 2 h prior to exposure to thapsigargin and mitochondrial calcium was determined with Rhod2 AM (Figure 4A, B) [18]. Pre-incubation with CsA increased mitochondrial calcium uptake in mutant cells exposed to thapsigargin (Figure 4A, B). Previous studies have shown that treatment with CsA or FK506 significantly reduced the ionophore-induced rise in cytosolic calcium and mitochondrial depolarization in both neurons and astrocytes [37]. Interestingly, in wild type cells, CsA reduced mitochondrial calcium increase induced by thapsigargin (compare both cell types in Figure 4B). These observations suggest that mPTP could play an active role in normal mitochondrial calcium uptake in striatal cells.

### Ruthenium red failed to prevent mitochondrial dysfunction induced by pathological calcium increases in clonal striatal cells

Ruthenium red, an inhibitor of the mitochondrial calcium uniporter, that prevents mitochondrial calcium uptake [11], rescued mutant striatal cells from cell death and mitochondrial depolarization induced by the treatment with the mitochondrial toxin 3-nitropropionic acid (3-NP) [11]. Therefore, we tested the effect of ruthenium red on the thapsigargin-induced loss of calcium uptake and depolarization by the mitochondria in mutant cells (Figure 4C and D). Figure 4C shows representative plots of mitochondrial calcium levels in striatal cells pre-treated with ruthenium red and exposed to 1 μM thapsigargin. In mutant striatal cells the treatment with ruthenium red treatment did not prevent the reduction of mitochondrial calcium uptake (Figure 4C, D). In contrast, ruthenium red prevented the mitochondrial calcium uptake induced by thapsigargin in wild type cells, suggesting the maintenance of a normal mitochondrial calcium regulation (Figure 4C, D). More importantly, pre-treatment with ruthenium red did not prevent mitochondrial depolarization induced by calcium stress in mutant huntingtin cells (data not shown). These data indicate that the mitochondrial calcium uniporter is not involved in the mitochondrial dysfunction observed in mutant huntingtin cells exposed to calcium stress.

### Cyclosporine A prevented Reactive Oxygen Species (ROS) production and mMTP opening induced by thapsigargin in mutant huntingtin cells

**Figure 5:**
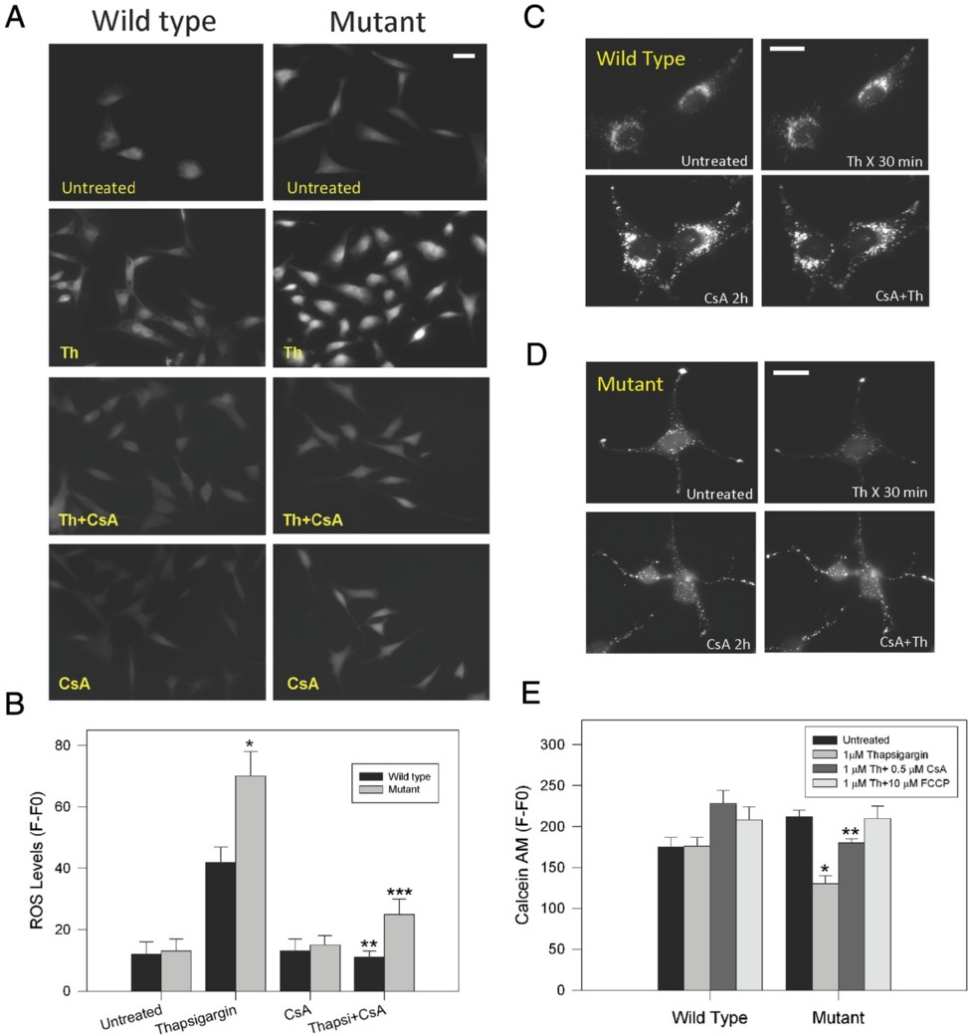
**Cyclosporine A prevented Reactive Oxygen Species (ROS) production and mMTP opening induced by thapsigargin in mutant huntingtin cells. A**, representative fluorescence images of 2,7-dichlorofluorescein indicating the levels of ROS striatal cells challenged with 1 μM thapsigargin (Th) for 1 h, CsA for 2 h and both. Increased levels of ROS in striatal cells treated with thapsigargin **(A** and **B)** were prevented by pretreatment with CsA. Bar =10 μm. **B**, Quantitative ROS levels. Data are the mean ± S.E.M. of 3 independent experiments. *, *p* < 0.05 compared with thapsigargin treated wild type cells; **, *p* < 0.05 compared with thapsigargin treated wild type cells. ***, *p* < 0.05 compared with mutant cells treated with thapsigargin. *p* < 0.05 by non-paired Student’s *t* test. **C** and **D**, representative fluorescence images of wild type **(C)** and mutant cells **(D)** cells loaded with calcein AM/Cobalt for 30 min indicating the state of the mPTP [35]. Cells were treated with 1 μM thapsigargin (Th) for 30 1 h and pre-treated with 0.5 μM CsA for 2 h. Treatment with thapsigargin decreased calcein fluorescence intensity in mutant cells indicating that calcium overload opens the mPTP **(C)**. Treatment with CsA prevented the loss of calcein intensity in mutant cells, closing the mPTP. In **C** and **D** bar represents 10 μM. **E**, Calcein intensity levels in striatal cells treated with indicated conditions for 1 h. Quantitation in treated mutant cells revealed that thapsigargin induced mPTP opening. CsA prevented mPTP opening in mutant cells exposed to thapsigargin. Mitochondria depolarization induced by FCCP did not affect mPTP in mutant huntingtin cells. Data correspond to the mean ± S.E.M. of 4 independent experiments. * *p* < 0.05 compared with wild type cells treated with thapsigargin. ** *p* < 0.05 compared with mutant cells treated with thapsigargin.

ROS production is often used as a measure of mitochondrial function [38-41]. Given that treatment with thapsigargin resulted in mitochondrial dysfunction in mutant cells, and that CsA prevented this impairment, we determined if thapsigargin affected ROS production in wild type and mutant cells. Cells were loaded with 2′,7′-dichlorodihydrofluorescein diacetate (2.7-DCF), a cell-permeant indicator of ROS, and treated with thapsigargin and CsA alone or in combination. Because there were a few reports that indicated some problems with the use of 2.7-DCF for ROS determinations [42], we performed control studies in where striatal cells loaded with 2.7-DCF were treated with 500 μM H_2_O_2_ for 30 min and changes in 2.7-DCF fluorescence intensity were evaluated by confocal microscopy (Additional file 4: Figure S4). Treatment with H_2_O_2_ produced an increase in ROS production that was consistently detected by 2.7-DCF (Additional file 4: Figure S4). Thapsigargin induced a 3 to 5-fold increase of ROS production in wild type and mutant cells (Figure 5A, B; Additional file 4: Figure S4B), reaching higher ROS levels in mutant than in wild type cells (Figure 5B and Additional file 1: Figure S4B) as detected with 2.7-DCF. To corroborate these findings, we evaluated superoxide levels induced by thapsigargin using the MitoSOX™ (MitoSOX) in the same experimental paradigm (Additional file 4: Figure S4C). Mutant cells consistently produced more superoxide than wild type cells in response to thapsigargin treatment (Additional file 4: Figure S4C). Interestingly, CsA treatment significantly prevented the ROS production induced by calcium stress in both cell types (Figure 5B). Quantitated data of 2.7-DCF fluorescence intensity from 4 independent experiments shows a significant increase in ROS production in the mutant cells compared with wild type cells (Figure 5B). This increase in ROS production was prevented by CsA treatment (Figure 5B) suggesting that inhibition of mPTP by CsA reduced ROS levels in mutant huntingtin expressing cells exposed to pathological calcium overload.

The mPTP is defined by its inhibition by CsA, which binds to mitochondrial cyclophilin D and blocks the calcium-induced mPTP opening [35,36,43]. Opening of mPTP causes the mitochondrial inner membrane (MIM) to become permeable and leads to the loss of the ions concentration gradients and dissipation of the mitochondrial potential [31,32]. To determine if the mPTP was open in mutant cells exposed to calcium overload, we used the cobalt/calcein AM quenching method (Figure 5C-E) [35,44]. In mutant cells treated with thapsigargin, cobalt quenched calcein fluorescence inside the mitochondria due to free movement of these molecules through an open mPTP, decreasing by 35% calcein fluorescence intensity (Representative images in Figure 5C and quantitative data in Figure 5E). However, when mutant cells were treated with thapsigargin plus CsA, mitochondrial calcein fluorescence was retained, suggesting that CsA closed the mPTP, preventing cobalt entry (Representative images in Figure 5D and quantitative data in Figure 5E). As a control, we evaluated cobalt quenched calcein intensity in striatal cells treated with thapsigargin in the presence of 10 μM of the mitochondrial uncoupler FCCP (Figure 5E). FCCP treatment significantly prevented loss of calcein fluorescence from striatal cells mitochondria, indicating that mPTP has a strong dependency on pathological calcium increases (Figure 5E) [44].

To complement these results, we tested if the blocking of mPTP by CsA could have an effect on cell viability in striatal cells exposed to calcium stress (Additional file 5: Figure S5). Striatal cells were treated with 1 μM thapsigargin (Th), or 1 μM thapsigargin + 0.5 μM CsA (Th + CsA) for 24 h and cell viability was estimated with calcein AM (green) and Propidium Iodide (PI) staining. Calcein staining represents cells that are metabolically active and PI staining indicates a loss of the nuclear membrane integrity [11]. Treatment with thapsigargin significantly decreased cell viability (calsein/PI fluorescence ratio) in mutant huntingtin cells (Additional file 6: Figure S6). Inhibition of mPTP opening with CsA prevented cell viability loss induced by thapsigargin in mutant cells. These findings indicated that pathological calcium stress could affect mitochondria function, and then compromise cell viability in mutant huntingtin cells, through the opening of mitochondrial permeability transition pore.

### Blockage of mMTP prevented mitochondrial fragmentation induced by thapsigargin in mutant huntingtin cells

**Figure 6:**
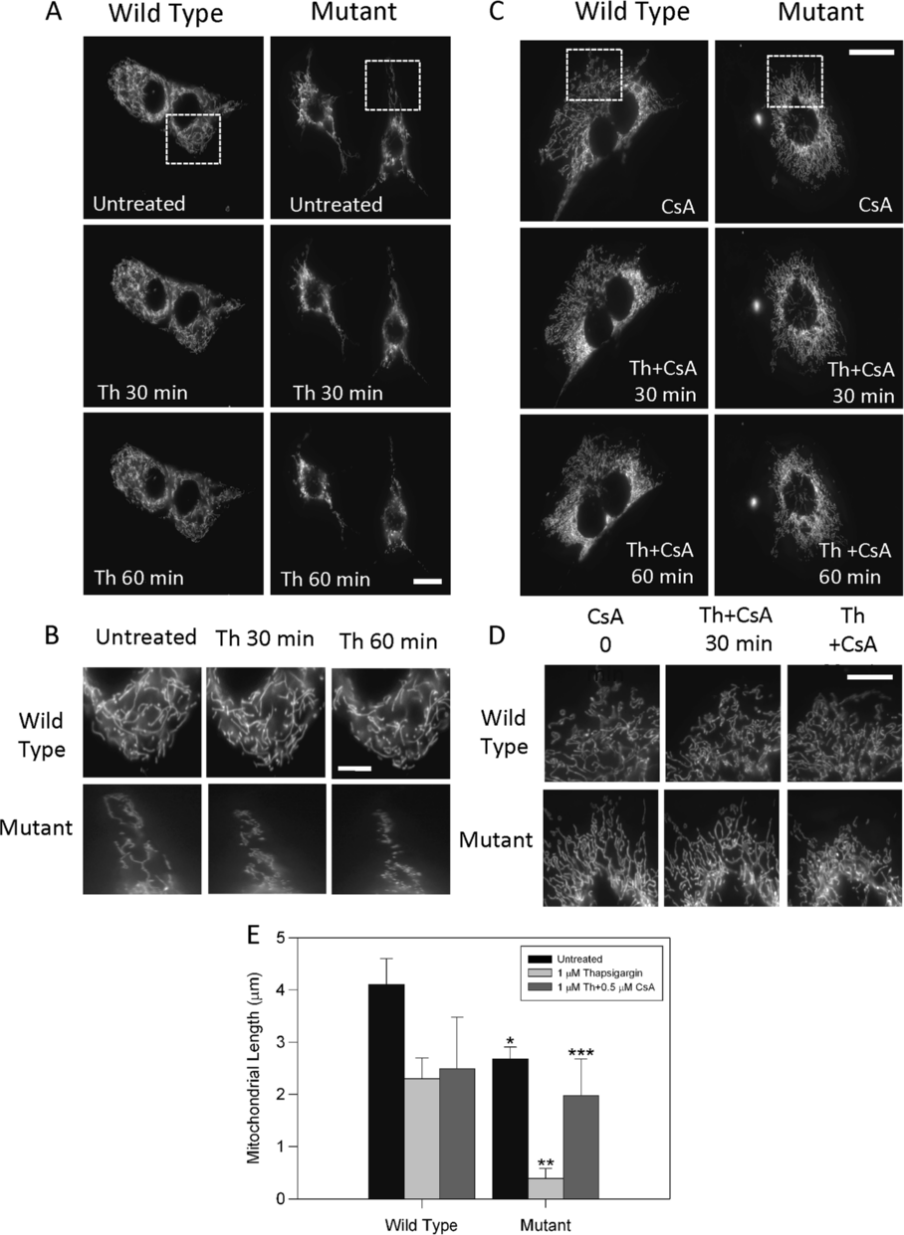
**Mitochondrial fragmentation induced by calcium overload was prevented by inhibition of mPTP in mutant huntingtin cells. A**, striatal cells transfected with Mito-GFP protein were treated with 1 μM thapsigargin (Th) during 60 min before live fluorescence images were taken. Images were acquired from full resolution images obtained with 63X objective in a Zeiss Axiovert LED fluorescence microscope. Thapsigargin treatment induced a significant fragmentation of mitochondria in mutant compared with wild type cells. Bar = 10 μm. **B**, magnification of boxed regions from **A** to emphasize differences of mitochondrial morphology in striatal cells. Bar = 5 μm. **C**, striatal cells transfected with Mito-GFP protein were treated with thapsigargin (Th) plus cyclosporine A (CsA) during 60 min and live fluorescence images were obtained. Images were acquired from full resolution images obtained with 63X objective in a Zeiss Axiovert LED fluorescence microscope. Thapsigargin treatment induced significant fragmentation of mitochondria in mutant compared with wild type cells. Mitochondria fragmentation produced by calcium stress was abolished by mPTP closure induced by 0.5 μM CsA treatment for 2 h. Bar = 10 μm. **D**, magnification of boxed regions from **A** to emphasize differences of mitochondrial morphology in striatal cells. Bar = 5 μm. **E**, quantitation of mitochondrial length of 4 independent experiments showed significant mitochondria fragmentation in mutant cells exposed to thapsigargin and this effect was prevented by co-treatment with CsA. Data correspond to the mean ± S.E.M. of 4 independent experiments. * *p* < 0.05 compared with wild type treated with thapsigargin. ** *p* < 0.05 compared with mutant cells treated with thapsigargin.

Mitochondria are morphologically dynamic organelles distributed throughout the cell by fission and fusion to form individual units or interconnected networks [25,26,45]. In normal healthy cells, there is equilibrium between mitochondria fusion and fission [46,47]. Evidence from experimental models suggests that mitochondrial fission and fusion appears to be altered in HD, contributing to neuronal dysfunction and death [21,22,45,48]. Mutant huntingtin appears to physically impair mitochondrial mobility and trafficking [21]. Increasing length of polyglutamine repeats length in mutant huntingtin constructs expressed in HeLa cells results in greater mitochondrial fragmentation and reduced ATP levels [22]. More importantly, we showed that in basal conditions mutant cells displayed more fragmented mitochondria than wild type striatal cells, and that this effect was prevented by the increase of Nrf2 expression, a transcriptional factor key in the regulation of ROS defense mechanism [24]. Mitochondrial fragmentation was accompanied with alterations on the expression levels of Drp1 and Opa1, key regulators of mitochondrial fission and fusion [24]. To complement these previous findings, we evaluated if calcium overload affects mitochondrial morphology of clonal striatal cells expressing mutant huntingtin. To evaluate changes in mitochondrial morphology, wild type and mutant striatal cells were transfected with a Mito-GFP construct and were mounted in a microscope chamber for time-lapse studies 24 h later. Mitochondrial morphology changes were evaluated for striatal cells treated with thapsigargin and thapsigargin plus CsA (Figure 6). Figure 6 shows representative images of mitochondrial morphology of wild type and mutant cells expressing Mito-GFP treated with thapsigargin for 1 h. As previously shown, mitochondria from mutant cells were more fragmented than those from wild type cells (Figure 6A, B) [24] and pathological increase in calcium induced by thapsigargin enhanced mitochondrial fragmentation in mutant cells compared with wild type cells (Figure 6A, B). Next, we assessed the effect of the pharmacological blockage of mPTP with CsA on mitochondrial morphology in striatal cells treated with thapsigargin (Figure 6C, D, E). Striatal cells were pre-treated with CsA for 2 h prior to thapsigargin treatment and their evaluation by time-lapse (Figure 6C, D, E). CsA reduced mitochondrial fragmentation induced by calcium overload in mutant huntingtin cells (Figure 6C, D, E), further suggesting that mPTP could play a role in mitochondrial impairment induced by calcium in mutant huntingtin cells.

### Mutant huntingtin expression induced mitochondrial impairment in cortical neurons exposed to pathological increases in calcium

**Figure 7:**
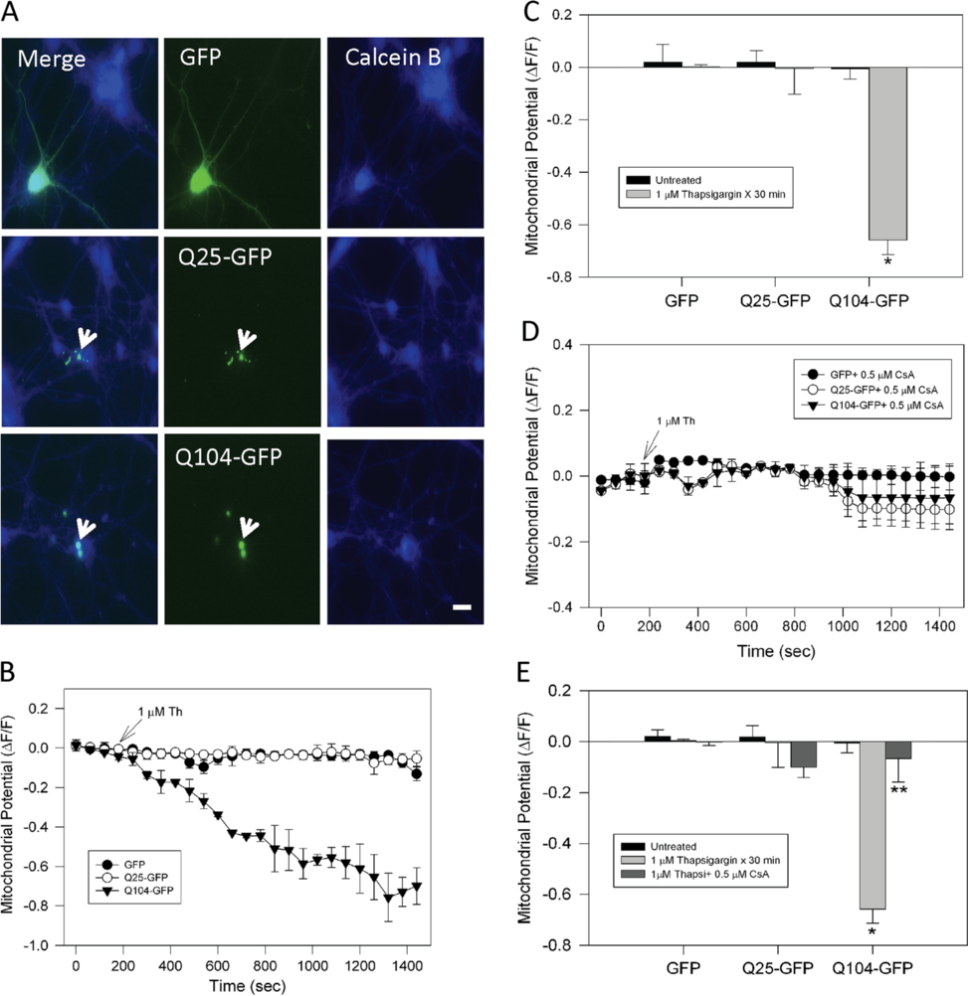
**Mitochondrial impairment induced by thapsigargin was prevented by cyclosporine A in neurons that expressed mutant huntingtin. A**, representative fluorescence images of cortical neurons loaded with calcein blue AM (Calcein B) and transfected with GFP alone, Q25-GFP (normal huntingtin), or Q104-GFP (mutant huntingtin). Normal and mutant huntingtin expression is indicated by GFP fluorescence intensity (white arrows) and calcein blue staining reveals neuronal morphology. Bar = 10 μm. **B**, transfected neurons were loaded with MitoRed for determination of mitochondrial potential. Representative trends show that the expression of Q104-GFP significantly reduced mitochondrial potential during treatment with 1 μM thapsigargin (Th). **C**, quantitation of mitochondrial potential levels showed significant mitochondrial damage in neurons that expressed Q104-GFP. Data correspond to the mean ± S.E.M. of 4 independent experiments. *, *p* < 0.05 compared with Q25-GFP neurons treated with thapsigargin. **D**, neurons transfected with GFP, Q25-GFP, or Q104-GFP were treated with 0.5 μM CsA for 2 h before mitochondria potential measurements. Representative trends show that the inhibition of mPTP by CsA prevented mitochondria injury induced by thapsigargin in Q104-GFP positive neurons. **E**, quantitation of mitochondria potential levels shows that CsA treatment significantly prevented mitochondrial impairment in neurons expressing Q104-GFP compared with the other conditions. Data correspond to the mean ± S.E.M. of 4 independent experiments. * *p* < 0.05 compared with Q25-GFP neurons treated with thapsigargin. ** *p* < 0.05 compared with Q104-GFP neurons treated with thapsigargin.

Here, we have observed that in clonal striatal cells, a cell model expresses physiological levels of mutant huntingtin, that calcium overload induced mitochondrial impairment and oxidative stress. Therefore, we expressed different polyglutamine versions of exon 1 of the huntingtin protein in primary cortical neurons. Q25-GFP was considered representative of normal huntingtin protein, whereas Q104-GFP was used to mimic the effects of mutant huntingtin. Figure 7A shows representative images from cortical neurons transfected with GFP (control vector), Q25-GFP and Q104-GFP labeled with calcein blue as a neuronal morphology indicator. Transfected neurons were loaded with MitoRed to observe mitochondrial potential changes in response to calcium stress (Figure 7B). As previously observed in mutant striatal cells, calcium stress induced a significant mitochondrial membrane potential loss in cortical neurons expressing Q104-GFP (Figure 7B, C, and representative images in Additional file 6: Figure S6). Representative graphs and quantitated studies showed that thapsigargin rapidly reduced mitochondrial membrane potential (Figure 7B, C).

### Blockage of mitochondrial permeability transition pore (mPTP) prevented mitochondrial dysfunction induced by thapsigargin in cortical neurons expressing mutant huntingtin

The effect of CsA on mitochondrial membrane potential changes induced by calcium overload was evaluated in cortical neurons expressing GFP, Q25-GFP, or Q104-GFP (Figure 7D, E). Cortical neurons transfected with GFP or Q25-GFP did not show significant changes in mitochondrial membrane potential in response to thapsigargin treatment with or without CsA pre-treatment (Figure 7D, E). More importantly, CsA abolished mitochondrial membrane potential loss induced in cortical neurons that expressed Q104-GFP, further strengthening our proposal that mPTP plays an important role in mitochondrial dysfunction induced by calcium stress in mutant huntingtin expressing cells (Figure 7D, E).

Altogether, these results indicate that expression of mutant huntingtin could have a negative effect on mitochondria function, reducing their capacity to respond to calcium stress. This effect is likely mediated by mPTP opening in mutant huntingtin expressing cells, resulting in an increased oxidative stress, calcium handling defects, mitochondrial fragmentation and finally in neuronal dysfunction.

## Discussion

**Figure 8:**
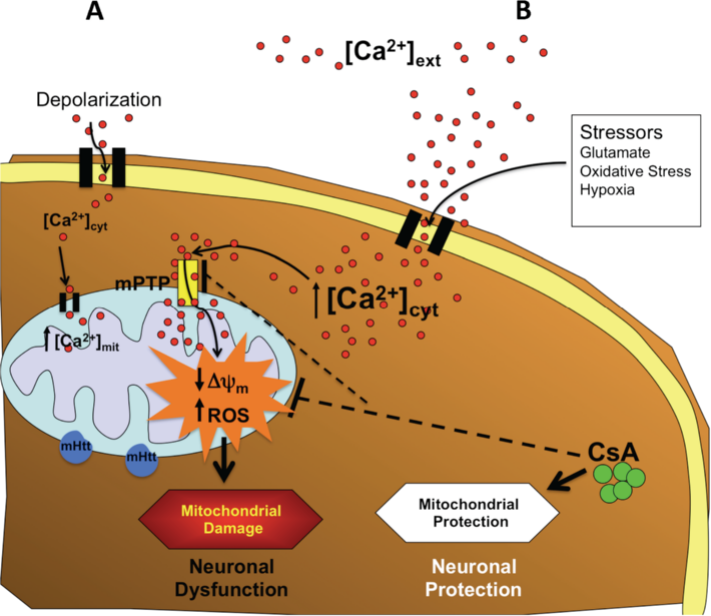
**Role of mPTP in mitochondrial damage induced by mutant huntingtin.** Representative scheme showing the interplay between calcium regulation, mitochondrial function and mPTP in mutant huntingtin cells. **(A)**, in response to controlled cytosolic calcium increase striatal cells can efficiently regulate calcium homeostasis and mitochondrial function even with the presence of mutant huntingtin. However, pathological calcium increases activated mPTP inducing mitochondrial depolarization and oxidative stress conducing to neuronal dysfunction **(B)**. These two signs of mitochondrial damage can be prevented by inhibition with CsA, an inhibitor of mPTP, which improve mitochondrial function and neuronal health.

Our principal finding is that mutant huntingtin affects mitochondrial function by modifying mitochondrial calcium homeostasis, mitochondrial morphology and ROS handling through opening of mPTP. These mitochondrial impairments in mutant huntingtin-expressing cells only occurred after calcium overload, simulating pathophysiological conditions that may occur in HD (Figure 8). CsA, a well-accepted inhibitor of mPTP opening, prevented these events, further supporting the participation of mPTP in the mitochondrial dysfunction (Figure 8). In addition, CsA prevented cell viability loss induced by calcium stress in mutant huntingtin cells (Additional file 5: Figure S5), confirming the importance that mitochondrial impairment have in the striatal neuronal dysfunction. Interestingly, we showed that huntingtin protein colocalized with MitoRed (mitochondrial marker) and cytochrome c in untreated striatal cells (Additional file 1: Figure S1). Also, previous studies of mitochondria subfractionation indicated that huntingtin was associated with the outer mitochondrial membrane in striatal cells [20]. Therefore, it is possible that binding of mutant huntingtin with mitochondria sensitizes this organelle to calcium stress leading to mPTP opening. Interestingly, mitochondrial injury induced by mutant huntingtin in response to calcium stress was replicated in cortical neurons transfected with mutant constructs containing an expanded polyglutamine domain (Figure 7), confirming that observations presented in this study support previous findings in where mutant huntingtin induced mitochondrial calcium handling defects in isolated mitochondrial from striatal cells [17-19]. From these and other findings we conclude that mitochondrial dysfunction in mutant cells could be triggered by cumulative cytosolic calcium increases and that these effects could contribute to the progression of striatal neuronal death reported in HD [49] (Figure 8).

It has been shown that elevated calcium can potentiate cell death-related events, including mPTP opening [30,50]. Interestingly, CsA prevented the reduction in mitochondrial calcium uptake, mitochondrial potential loss, and cell viability loss in mutant cells exposed to thapsigargin; in contrast to ruthenium red, which did not show any protective effect, indicating that regular mitochondrial calcium uptake mediated by uniporter is not involved in mitochondrial impairment induced by mutant huntingtin. In addition, FK-506, an inhibitor of calcineurin activity, had no significant effect on mitochondrial dysfunction induced in mutant striatal cells by calcium overload, indicating that CsA is specifically acting on mPTP opening [35].

In addition, we observed that treatment with H_2_O_2_ and FCCP induced a similar mitochondrial impairment in both wild type and mutant huntingtin cells, and this effect was not prevented or ameliorated by CsA treatment (Figure 2D). Our observations support an interesting work recently published by Wang et al., showing that YAC128 mouse embryonic fibroblasts that express mutant huntingtin exhibit a high level of mitochondrial Ca^2+^ load and elevated superoxide levels compared with wild type cells [34]. Increased mitochondrial oxidative stress was dependent on mitochondrial Ca^2+^ overload in HD cells, because blocking mitochondrial Ca^2+^ uptake prevented elevated superoxide production [34]. In general, these observations are complementary with the evidence presented here and indicate that mitochondrial dysfunction triggered by mutant huntingtin is dependent on pathological calcium deregulation, an event that could be responsible for mPTP opening in mutant cells [51,52].

Previous evidence suggests that a toxic gain of function of cyclophilin D (CyP-D) could be responsible of mitochondrial impairment induced by calcium [30-32]. For example, studies in striatal mitochondria preparations show an increased sensitivity to calcium stress compared with cortical mitochondria, which was partially prevented by CsA [52]. In addition, Shirendeb et al. evaluating the mRNA levels of mitochondrial structural genes showed that CyP-D was upregulated in HD patients, and this upregulation increased as HD progressed [26]. These observations suggest that mPTP could be playing an active role in mitochondrial function in striatal cells and further supports its involvement in mitochondrial dysfunction mechanisms previously reported in HD.

Interestingly, we observed that mutant striatal cells transfected with Mito-GFP and exposed to thapsigargin showed an increased mitochondrial fragmentation compared with wild type cells (Figure 6). More importantly, pre-treatment with CsA prevented mitochondrial fragmentation in mutant striatal cells treated with thapsigargin, in agreement with previous reports showing that expression of increasing polyglutamine repeats results in greater mitochondrial fragmentation and reduced ATP levels in HeLa cells [22]. Our observations complement previous observations where striatal calbindin-positive neurons in HD patients showed a significant and progressive reduction in the number of mitochondria [23]. Also, we recently examined, changes in mitochondrial dynamics and structure in wild type and mutant cells in response to oxidative stress [24]. Mutant huntingtin cells display more fragmented mitochondria than wild type cells, concurrent with alterations in the expression levels of dynamin-related protein 1 (Drp1) and Opa1, key regulators of mitochondrial fission and fusion, respectively [24]. Lastly, mitochondrial PCR array studies using HD caudate nucleus specimens showed an increased mRNA expression of Drp1, fission 1 (Fis1), and decreased expression of: mitofusin1 (Mfn1), mitofusin 2 (Mfn2), and Opa1 relative to the control individuals [23]. These are proteins involved in mitochondrial localization, membrane translocation and polarization and transport that paralleled mitochondrial impairment [23,26]. Therefore, our findings in conjunction with previous evidence reveal that both mitochondrial function and altered mitochondrial morphogenesis could play an important role in the pathogenesis of HD.

Mice lacking of CyP-D a key regulator of mPTP, are resistant to oxidative stress and mitochondrial toxicity mediated by calcium [53], indicating that striatal mitochondria could be selectively more vulnerable to the mPTP opening. Despite our results suggesting that mitochondrial dysfunction could be mediated by mPTP opening in HD, it was recently shown that increased neuronal mitochondria calcium retention by CyP-D failed to ameliorate both behavioral and neuropathological features of R6/2 mice [51]. R6/2 mice are the most widely used HD study models because of its relatively rapid-onset and robust phenotype [54,55]. Even though the phenotype and neuropathology observed in R6/2 lines replicate several features observed in HD patients, R6/2 mice may not model critical aspects of the pathogenic process in HD [51]. Another important consideration is that negative outcome of CyP-D ablation could be due to compensatory mechanisms during development, which could affect mitochondrial function [51]. In addition, parameters like ROS production, mitochondrial membrane potential and mitochondrial fragmentation were not evaluated by Perry et al., [51], compared with our studies in where we evaluated all parameters mentioned above. More importantly, previous studies of our group showed a decreased expression of CyP-D protein levels in mutant huntingtin cells compared to wild type [56]. Therefore, it is plausible that greater association between mutant huntingtin and mitochondria (Additional file 1: Figure S1) facilitates mPTP opening by calcium stress and this could ultimate affects mitochondria function. However, additional experiments are needed to determine if the interaction of mutant huntingtin with other mitochondrial proteins could modify protein structures relevant for mPTP opening and thus contribute to the pathogenesis of HD.

## Conclusions

Taken together, our observations indicate that mutant huntingtin affects mitochondrial and neuronal function through mPTP opening and that this action must be trigger by accumulative calcium overload in striatal cells and cortical neurons. mPTP may affect mitochondrial calcium homeostasis, ROS production, and mitochondrial fusion/fission which contributes to the dysfunction of mitochondria in striatal cells, and thus likely is an important factor in the neurodegenerative processes that occur in HD brain.

## Methods

### Materials and plasmids

Chemicals, culture media and serum were obtained from Sigma-Aldrich (St. Louis, MO, USA), Roche (Alameda, CA, USA), and Invitrogen (Carlsbad, CA, USA). Fluo-3 AM, Rhod-2 AM, thapsigargin, Mitotracker Green FM (MTG), Mitotracker Red® CM-H_2_XRos (MitoRed), MitoSOX™ (MitoSOX), cyclosporine A (CsA), FK 506, KCl, calcein AM (green), calcein AM (blue), Propidium Iodide (PI), cobalt chloride, FCCP, DAPI, and 5-(and-6)-chloromethyl-2′,7′-dichlorodihydrofluorescein diacetate, acetyl ester (2,7-DCF) were obtained from Molecular Probes (Eugene, OR, USA). Anti-huntingtin antibody (mAB2166) was obtained from Chemicon International (Temecula, CA, USA) and anti-cytochrome C was obtained from BD Biosciences (San Jose, CA, USA). Q25-GFP and Q104-GFP constructs were kindly provided by Dr. Mathieu Lesort (UAB, AL, USA). Q25-GFP and Q104-GFP contain the N-terminal and poly Q region of huntingtin fused with GFP. Mito-GFP was kindly provided by Dr. Yisang Yoon (Medical College of Georgia, GA, USA).

### Cell culture

#### Striatal cells culture

Conditionally immortalized striatal progenitor cell lines STHdh^Q7/Q7^ expressing endogenous wild type huntingtin and STHdh^Q111/Q111^ cells expressing comparable levels of mutant huntingtin with 111 glutamines [27] were used. These cell lines, a generous gift from Dr. M. E. MacDonald, were prepared from wild type and homozygous Hdh^Q111/Q111^ knock-in mice as previously described [27]. Culture conditions were the same as described in previous studies [18,19,56].

#### Primary neuron culture and transfection

Primary cortical neuronal cultures from rat embryonic forebrains were prepared as previously described with some modifications [57]. Briefly, the brain of rats at embryonic day 17– 18 was obtained. Cortices were dissected, treated with 0.05% trypsin at 37°C for 30 min, and gently triturated with a fire polished glass Pasteur pipette. Cells were plated onto poly-D-lysine-coated coverslips or glass-bottom dishes in Minimum Essential Media (MEM) with 5% fetal bovine serum, 100 U/mL penicillin, and 100 μg/mL streptomycin. After three hours of seeding, medium was replaced with Neurobasal medium (NBM) supplemented with 0.4 mM glutamine and B27. To prevent proliferation of glial cells and astrocytes, neuronal cultures were treated with 2 μM AraC for 24 h. Half of the medium was replaced with fresh neurobasal medium supplemented with 0.4 mM glutamine and B27 every 3 days. All materials for cell culture were from Invitrogen, CA, USA.

Neurons were transiently transfected with 0.8 μg of plasmids using Lipofectamine 2000 (Invitrogen) at 7 days in vitro (DIV) following manufacturer protocols. Imaging experiments were performed at 9 DIV in a confocal microscope.

### Cell viability

Cell viability was determined by using the calcein-AM (green) and Propidium Iodide (PI) (red) assay with some modifications [11]. Briefly, non-fluorescent calcein-AM is converted into green fluorescent calcein by intracellular esterases and indicates the presence of active cell metabolism. PI binds to nucleic acid generating a fluorescence signal that indicates disruption of nuclear membrane. After treatment, striatal cells were incubated with calcein-AM (1 μM) and PI (5 μM) for 30 min at 37°C and then live fluorescence images were taken in a fluorescence microscope (Axiovert Colibri, Zeiss). Quantification of fluorescence intensity was made using Image Pro 6 software and cell viability was expressed as a calcein/PI ratio of the signal obtained from the cells untreated and exposed to indicate conditions.

### Intracellular Reactive Oxygen Species (ROS) measurement

Clonal striatal cells were plated on poly-L-lysine-coated coverslips (30,000 cells/coverslip) and treated with 1 μM thapsigargin, in absence or presence of 0.5 μM CsA for 2 h. After treatment, cells were incubated with the fluorescent probe 2, 7-DCF (10 μM) in Krebs-Ringer-Hepes (KRH) buffer supplemented with 5 mM glucose for 30 min [19,58]. Coverslips were washed two times with PBS solution and fixed with 4% *p*-formaldehyde for 5 min. Cells were photographed using a Zeiss Axiovision fluorescence microscope integrated with a CCD digital cool camera (Zeiss, Germany). Photographs were taken adjusting the same exposure time and gain detector in order to diminish the photobleaching of 2,7-DCF. Images quantification for each separate experiment was carried out analyzing 30 images (10-15 cells per image) for every indicated condition using Image-Pro Plus 6 software. Additionally, superoxide levels were evaluated using MitSOX™ dye in striatal cells treated with 1 μM thapsigargin during 1 h [57]. Cells grown on 35-mm dishes were incubated with 20 μM Mitotracker Green™ (MTG) and 200 nM MitoSOX Red for 20 min in KRH buffer supplemented with 5 mM glucose [19]. MTG accumulates in the lipophilic environment of live mitochondria, and it has been shown that its signal is independent of the mitochondrial potential [19]. Images were acquired using a 488-nm argon laser to excite MTG and a 563-nm He-Ne laser to excite MitoSOX Red fluorescence. Estimation of mitochondrial superoxide production was quantified using Image-Pro Plus 6 software. Results in intensity units were expressed as the average of fluorescence signal (F) minus background fluorescence (F0) in every image. Results in intensity units were expressed as average of fluorescence signal (F) minus background fluorescence (F0) in every image [19,58].

### Determination of mitochondria potential in live cells

Mitochondria membrane potential was determined using the mitochondrial dye, Mitotracker® Red CM-H_2_XRos (MitoRed) [18,19,57]. The validity of MitoRed as a reliable mitochondrial membrane potential indicator was previously defined in Milakovic et al., 2006 and Quintanilla et al., 2008 [18,19]. Striatal cells were loaded separately with MitoRed and TMRM and then were treated with the mitochondrial uncoupler FCCP (10 μM). Changes in intensity were recorded using a confocal microscope [18]. Both indicators presented equal changes in mitochondrial potential levels after FCCP treatment [18]. In addition, mitochondrial potential was evaluated in striatal cells treated with 1 nM 4-BrA23187 + 6 mM Ca^2+^ in the presence of 10 μM FCPP and mitochondrial potential was evaluated (Additional file 3: Figure S3). The treatment with 1 nM 4-BrA23187 did not induce a significant change in mitochondrial potential, mitochondrial calcium uptake, and cell viability [18]. Mutant and wild type presented equal mitochondrial potential loss after treatment (Additional file 3: Figure S3). Striatal cells were grown on poly-L-lysine-coated coverslips and cultured for 4 days. The cell cultures were then loaded for 30 min with MitoRed in KRH buffer supplemented with 5 mM glucose and 0.02% pluronic acid. Cells were then mounted in a chamber on a confocal laser scanning microscope (Leica SP2, Germany). Fluorescence changes determined by MitoRed fluorescence were used as a measure of mitochondria potential [18,19,57]. Signal from control cells and cells treated with different stimuli were compared using identical settings for laser power, and detector sensitivity for each separate experiment. The images were analyzed with a LCS Leica confocal software (Germany) and recorded as the mean MitoRed fluorescence signal per live cell. For each experiment, we analyzed MitoRed intensity changes in 10-15 cells in average. Estimation of fluorescence intensity of MitoRed was presented as the pseudo-ratio (∆F/F_o_), which was calculated using the following formula: ∆F/F_o_ = (F-F_base_)/(F_base_-B), where F is the measured fluorescence intensity of the indicator, F_base_ is the fluorescence intensity before the stimulation, and B is the background signal determined from the average of areas adjacent to the cells [18,19,57].

### *In situ* evaluation of mitochondrial permeability transition pore (mPTP)

mPTP opening was evaluated in striatal cells previously loaded with 1 μM calcein AM (green) in presence of 1 μM cobalt chloride for 30 min before treatment with thapsigargin [35]. Quenching of free calcein by cobalt chloride allows observing mitochondrial integrity as an mPTP indicator [35,44]. Striatal cells loaded with calcein/cobalt chrolide were treated with thapsigargin for 1 h and images were taken using a confocal microscope. Loss of mitochondrial integrity or mPTP was evaluated from calcein fluorescence intensity levels obtained from time-lapse images [35]. For each independent experiment, we analyzed calcein AM intensity changes in 10-15 cells in average.

### Cytosolic and mitochondrial calcium measurements

Cells grown on poly-L-lysine-coated 25 mm coverslips (37°C) were loaded with 5 μM Fluo-3 AM, and 10 μM Rhod-2 AM in KRH-glucose containing 0.02% pluronic acid for 30 min. The fluorescence changes determined by Fluo-3 represent the cytoplasmic calcium changes [19], and Rhod-2 fluorescence indicates calcium changes in the mitochondria [19,59,60]. To estimate Rhod-2 fluorescence pattern in live mitochondria, we used MTG [19]. Fluorescence was imaged with a confocal laser-scanning microscope (Leica TCS SP2) using a 40× water immersion lens, as previously described [18]. Images were acquired using a 488-nm Argon laser to excite Fluo-3 fluorescence and a 563-nm He-Ne laser to excite Rhod-2 fluorescence. Signals were collected at 505–530 nm (Fluo-3) and at 590 nm (Rhod-2). Fluorescence background signal was subtracted from cell fluorescence measurements in every experiment. The fluorescence intensity variation was recorded from 10-20 cells on average per experiment. Estimation of fluorescence intensity of Fluo-3 and Rhod-2 were presented as a pseudo-ratio (∆F/F_o_), as previously described [19].

### Analysis of mitochondrial morphology

Mitochondrial morphology was analyzed as described previously with modifications [57]. Briefly, clonal striatal cells were transfected with Mito-GFP using Lipofectamine 2000 for 24 h and subsequently replaced with fresh media. We estimated a transfection efficacy of 35%. Striatal cells expressing Mito-GFP were treated with CsA for 2 h previous to thapsigargin treatment and time-lapse images were registered using fluorescence Zeiss microscope. Mitochondrial length was analyzed as previously shown [19,57]. Briefly, mitochondrial length was calculated using the measured perimeter of identified objects, in striatal cells positive for Mito-GFP staining using Image Pro 6 software (Media Cybernetics, MA). We analyzed mitochondrial population from 10-15 cells presented in each image that was taken. For quantification purposes, we measured around 15-18 images for experiment in each condition indicated [19,57].

### Statistical analysis

Results were expressed as the mean ± S.E.M., and were analyzed using Student’s *t* test, or paired *t* test as indicated. Differences were considered significant if *p* < 0.05 or p < 0.01, as indicated.

## Abbreviations

HD: Huntington’s disease; mPTP: Mitochondrial permeability transition pore; CsA: Ciclosporine A; ROS: Reactive oxygen species; MitoGreen: Mitotracker Green FM; MitoRed: Mitotracker Red® CM-H_2_XRos.

## Competing interests

The authors declare that they have no competing interests.

## Authors’ contributions

Conceived and designed experiments: RAQ GWJ. Performed the experiments: RAQ YNJ. Analyzed the data: RAQ RVB GWJ. Contributed reagents/materials/analysis tools: RAQ RVB GWJ. Wrote the paper: RAQ RVB GWJ. All authors read and approved the final manuscript.

## Supplementary Material

Additional file 1: Figure S1 Expression of huntingtin in clonal striatal cells. Striatal cells were grown at glass coverslips and then were fixed and stained with: anti-huntingtin antibody, anti-cytochrome antibody, DAPI, and the mitochondrial indicator MitoRed to study the levels and the localization of huntingtin. Confocal images reveal a high degree of colocalization between huntingtin and the mitochondrial markers cytochrome c and MitoRed. Bars = 10 μm. Click here for file

Additional file 2: Figure S2 Cytosolic calcium levels in striatal cells. **A**, representative confocal images of striatal cells loaded with Fluo3 AM. Thapsigargin treatment induced an acute and transient calcium elevation in both cell types. Bar =10 μm. **B**, representative trends of cytosolic calcium levels in striatal cells treated with 1 μM thapsigargin during 30 min. Data correspond to the mean ± S.E.M. of 4 independent experiments. **C**, quantitated data from 4 independent experiments of the peak of cytosolic calcium levels observed in every condition indicated. Data are mean ± S.E.M. * *p* < 0.05 compared with wild type cells treated with 60 mM KCL; ** *p* < 0.05 compared with mutant cells treated with 60 mM KCL; # *p* < 0.05 compared with wild type cells treated with 1 nM 4-BrA23187 + 6 mM Ca^2+^; ## *p* < 0.05 compared with mutant cells treated with 1 nM 4-BrA23187 + 6 mM Ca^2+^. Click here for file

Additional file 3: Figure S3 Treatment with 4-BrA23187 + 6 mM Ca^2+^ affects mitochondrial health in mutant cells. **A**, representative confocal images of striatal cells loaded with MitoRed to evaluate mitochondrial potential changes in response to 1 nM 4-BrA23187 and 1 nM 4Br-A23187 + 6 mM Ca^2+^ treatment for 30 min. **B**, quantitated data of striatal cells untreated and treated with 1 nM 4-BrA2387 + 6 mM Ca^2+^ and 1 nM 4-BrA23187 + 6 mM Ca^2+^ in the presence of 10 μM FCCP. Data are the mean ± S.E.M. of 3 independent experiments. *, *p* < 0.05 compared with wild type cells treated with 1 nM 4-BrA23187 + 6 mM Ca^2+^. Click here for file

Additional file 4: Figure S4 Calcium stress induced oxidative stress in mutant huntingtin expressing cells. A, representative trends of ROS levels induced by the treatment with 100 μM H_2_O_2_ during 30 min. B, representative trends of superoxide levels evaluated using MitoSOX/MTG (see Methods for details) in striatal cells exposed to 1 μM thapsigargin for 30 min. C, ROS levels were evaluated with 2.7-DCF in wild type cells exposed to 500 μM H_2_O_2_ for 30 min. Click here for file

Additional file 5: Figure S5 Treatment with cyclosporine A prevents cell viability loss in mutant huntingtin cells exposed to calcium overload. **A**, representative images from striatal cells untreated and treated with 1 μM thapsigargin (Th), and 1 μM thapsigarin plus 0.5 μM cyclosporine A (Th + CsA) for 24 h and loaded with calcein AM/Propidium Iodide (PI) to evaluate cell viability loss. Cell death was estimated calculating the ratio of fluorescence intensity between calcein (green) and PI (red) channels. Bars represent 10 μm. **B**, quantification of calcein/PI ratio from fluorescence images of 3 independent experiments. Thapsigargin induced a significant decrease in cell viability in mutant cells. CsA prevented cell viability loss induced by thapsigargin in mutant cells. Data are the mean ± S.E.M. of 3 independent experiments. *, ** *p* < 0.05, using Student’s *t* test. Click here for file

Additional file 6: Figure S6 Calcium stress induced mitochondrial impairment in cortical neurons expressing mutant huntingtin. **A**, **B**, **C**, representative confocal images of cortical neurons transfected with GFP, Q25-GFP, and Q104-GFP and loaded with MitoRed to measure mitochondrial potential changes in response to 1 μM thapsigargin. Treatment with thapsigargin did not change mitochondrial potential in GFP and Q25-GFP positive neurons (A, B). However, thapsigargin decreased mitochondrial potential levels in Q104-GFP loaded cells (C). White arrows indicate Q25-GFP and Q109-GFP expression in cortical neurons. Bar = 10 μm. Click here for file
