# Supplementary figures and images for: Mitochondrial permeability transition pore induces mitochondria injury in Huntington disease
Source: Mol Neurodegener. 2013 Dec 11;8:45. doi: 10.1186/1750-1326-8-45 (PMC3878840; doi:10.1186/1750-1326-8-45)

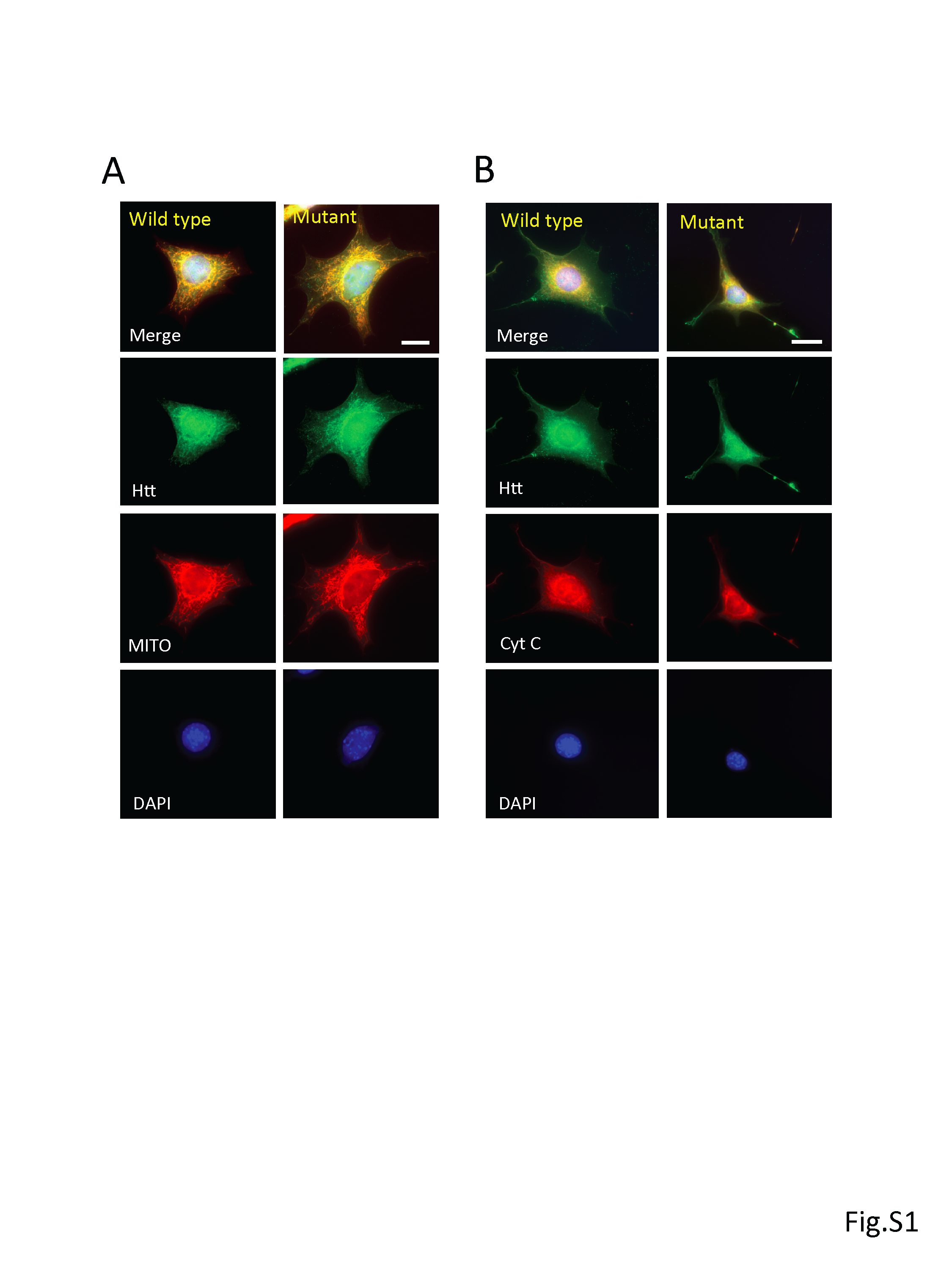

Supplement: Additional file 1: Figure S1 — Expression of huntingtin in clonal striatal cells. Striatal cells were grown at glass coverslips and then were fixed and stained with: anti-huntingtin antibody, anti-cytochrome antibody, DAPI, and the mitochondrial indicator MitoRed to study the levels and the localization of huntingtin. Confocal images reveal a high degree of colocalization between huntingtin and the mitochondrial markers cytochrome c and MitoRed. Bars = 10 μm. [file 1750-1326-8-45-S1.tiff]

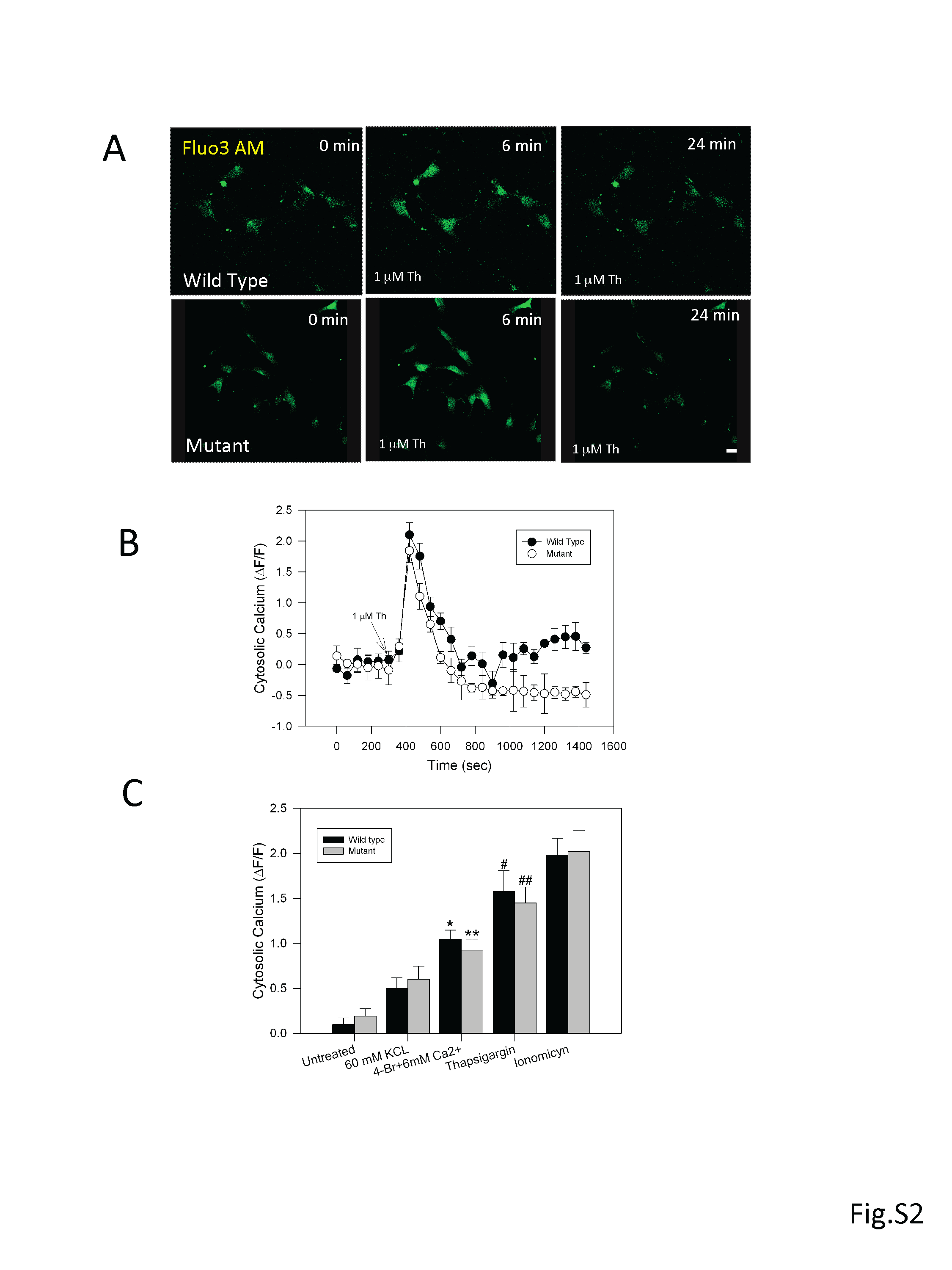

Supplement: Additional file 2: Figure S2 — Cytosolic calcium levels in striatal cells. A, representative confocal images of striatal cells loaded with Fluo3 AM. Thapsigargin treatment induced an acute and transient calcium elevation in both cell types. Bar =10 μm. B, representative trends of cytosolic calcium levels in striatal cells treated with 1 μM thapsigargin during 30 min. Data correspond to the mean ± S.E.M. of 4 independent experiments. C, quantitated data from 4 independent experiments of the peak of cytosolic calcium levels observed in every condition indicated. Data are mean ± S.E.M. * p < 0.05 compared with wild type cells treated with 60 mM KCL; ** p < 0.05 compared with mutant cells treated with 60 mM KCL; # p < 0.05 compared with wild type cells treated with 1 nM 4-BrA23187 + 6 mM Ca2+; ## p < 0.05 compared with mutant cells treated with 1 nM 4-BrA23187 + 6 mM Ca2+. [file 1750-1326-8-45-S2.tiff]

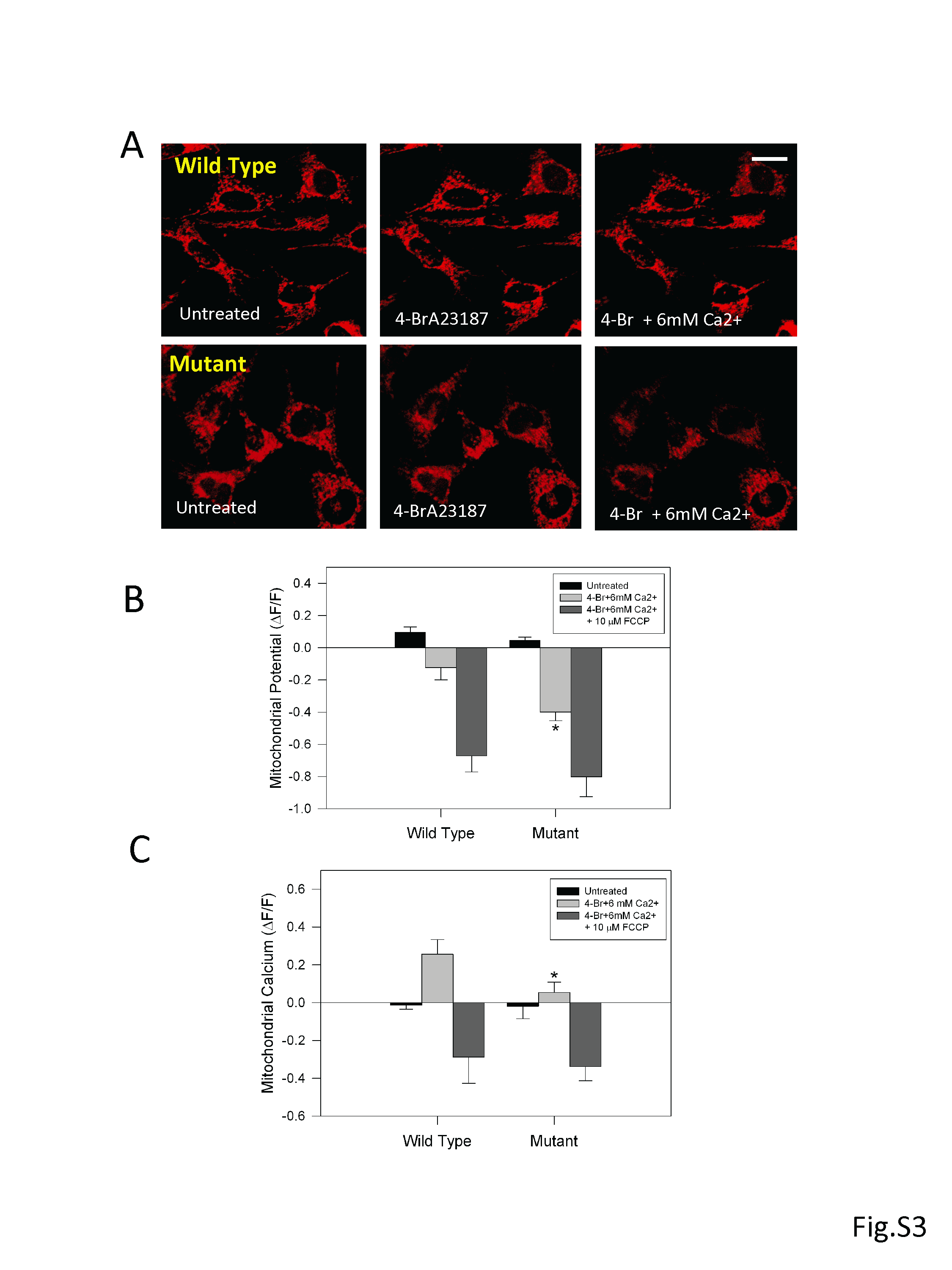

Supplement: Additional file 3: Figure S3 — Treatment with 4-BrA23187 + 6 mM Ca2+ affects mitochondrial health in mutant cells. A, representative confocal images of striatal cells loaded with MitoRed to evaluate mitochondrial potential changes in response to 1 nM 4-BrA23187 and 1 nM 4Br-A23187 + 6 mM Ca2+ treatment for 30 min. B, quantitated data of striatal cells untreated and treated with 1 nM 4-BrA2387 + 6 mM Ca2+ and 1 nM 4-BrA23187 + 6 mM Ca2+ in the presence of 10 μM FCCP. Data are the mean ± S.E.M. of 3 independent experiments. *, p < 0.05 compared with wild type cells treated with 1 nM 4-BrA23187 + 6 mM Ca2+. [file 1750-1326-8-45-S3.tiff]

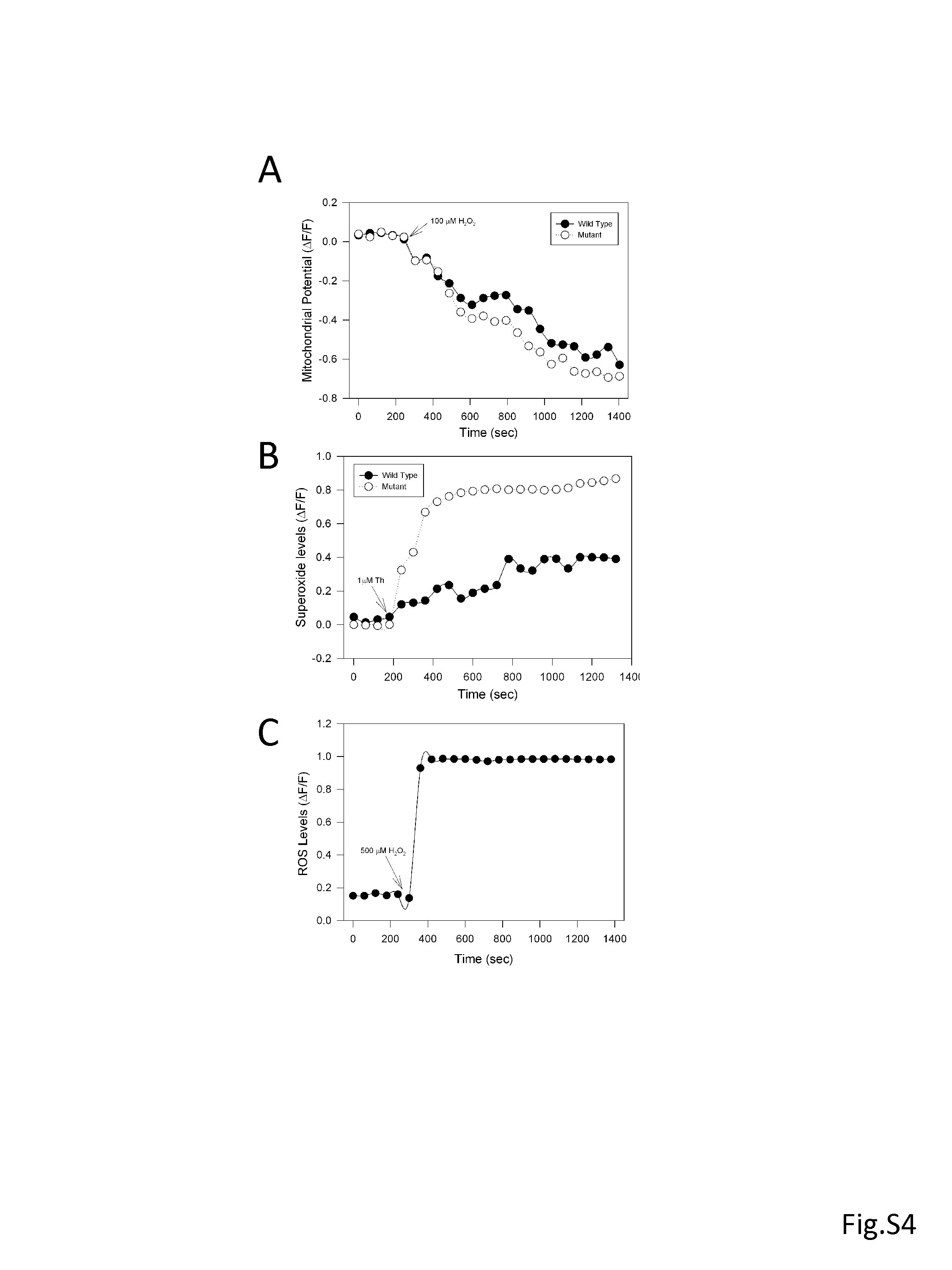

Supplement: Additional file 4: Figure S4 — Calcium stress induced oxidative stress in mutant huntingtin expressing cells. A, representative trends of ROS levels induced by the treatment with 100 μM H2O2 during 30 min. B, representative trends of superoxide levels evaluated using MitoSOX/MTG (see Methods for details) in striatal cells exposed to 1 μM thapsigargin for 30 min. C, ROS levels were evaluated with 2.7-DCF in wild type cells exposed to 500 μM H2O2 for 30 min. [file 1750-1326-8-45-S4.tiff]

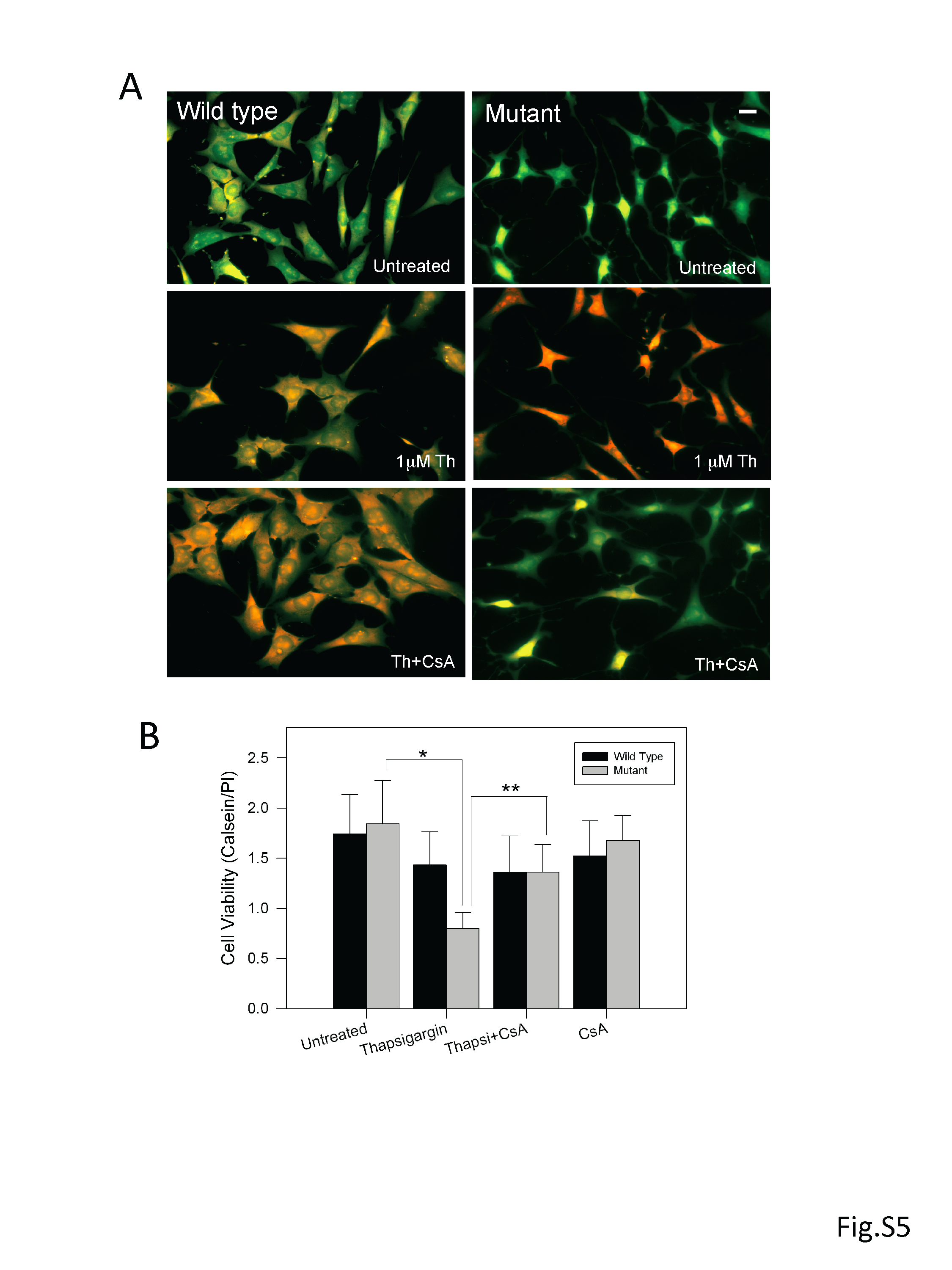

Supplement: Additional file 5: Figure S5 — Treatment with cyclosporine A prevents cell viability loss in mutant huntingtin cells exposed to calcium overload. A, representative images from striatal cells untreated and treated with 1 μM thapsigargin (Th), and 1 μM thapsigarin plus 0.5 μM cyclosporine A (Th + CsA) for 24 h and loaded with calcein AM/Propidium Iodide (PI) to evaluate cell viability loss. Cell death was estimated calculating the ratio of fluorescence intensity between calcein (green) and PI (red) channels. Bars represent 10 μm. B, quantification of calcein/PI ratio from fluorescence images of 3 independent experiments. Thapsigargin induced a significant decrease in cell viability in mutant cells. CsA prevented cell viability loss induced by thapsigargin in mutant cells. Data are the mean ± S.E.M. of 3 independent experiments. *, ** p < 0.05, using Student’s t test. [file 1750-1326-8-45-S5.tiff]

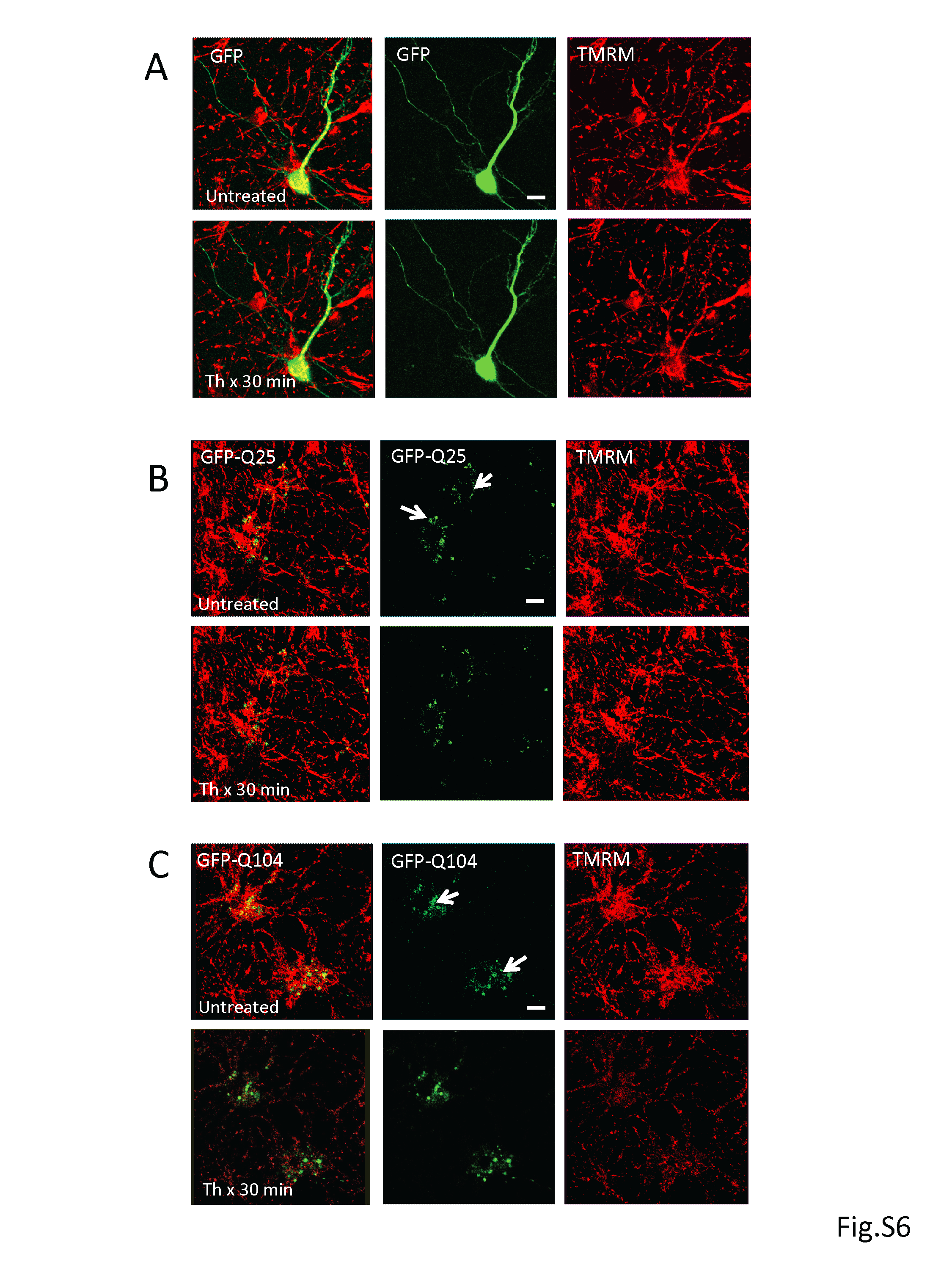

Supplement: Additional file 6: Figure S6 — Calcium stress induced mitochondrial impairment in cortical neurons expressing mutant huntingtin. A, B, C, representative confocal images of cortical neurons transfected with GFP, Q25-GFP, and Q104-GFP and loaded with MitoRed to measure mitochondrial potential changes in response to 1 μM thapsigargin. Treatment with thapsigargin did not change mitochondrial potential in GFP and Q25-GFP positive neurons (A, B). However, thapsigargin decreased mitochondrial potential levels in Q104-GFP loaded cells (C). White arrows indicate Q25-GFP and Q109-GFP expression in cortical neurons. Bar = 10 μm. [file 1750-1326-8-45-S6.tiff]
